# Curved Nanomagnets: An Archetype for the Skyrmionic States at Ambient Conditions

**DOI:** 10.1021/acs.nanolett.5c00773

**Published:** 2025-04-10

**Authors:** Danian A. Dugato, Wesley B. F. Jalil, Ramon Cardias, Marcelo Albuquerque, Marcio Costa, Trevor P. Almeida, Kayla Fallon, András Kovács, Stephen McVitie, Rafal E. Dunin-Borkowski, Flavio Garcia

**Affiliations:** † 74350 Centro Brasileiro de Pesquisas Físicas (CBPF), Rua Dr Xavier Sigaud 150, Urca, 22290-180, Rio de Janeiro-RJ, Brazil; ‡ Instituto de Física, 28110 Universidade Federal Fluminense, 24210-346 Niterói RJ, Brazil; ¶ SUPA, School of Physics and Astronomy, University of Glasgow, Glasgow G12 8QQ, United Kingdom; § Ernst Ruska-Centre for Microscopy and Spectroscopy with Electrons, 28334 Forschungszentrum Jülich, 52425 Jülich, Germany

**Keywords:** Skyrmion, Dzyaloshinskii−Moriya Interaction, Perpendicular Magnetic Anisotropy, Electron Holography, MFM, DFT

## Abstract

Stabilizing magnetic skyrmions is a critical issue in spintronics, impacting data storage and computing. This study investigates skyrmion and skyrmionium phenomena within a hexagonal array of curved nanomagnets. Utilizing atomistic calculations, micromagnetic simulations, and experimental methods such as magnetic force microscopy and electron holography, we analyze the interplay between magnetic parameters, curvature, and the interfacial Dzyaloshinskii–Moriya interaction (iDMI) in the formation of these structures. We observed that isolated skyrmions and mixed skyrmionic phases can spontaneously form in a symmetric Pt/Co/Pt multilayer curved nanomagnet matrix without external fields at room temperature. Our findings highlight the considerable influence of geometric curvature on iDMI, providing insights for engineering skyrmionic configurations. This research enhances our understanding of nanomagnetism and contributes to the advancement of skyrmion-based technologies.

## Graphical abstract

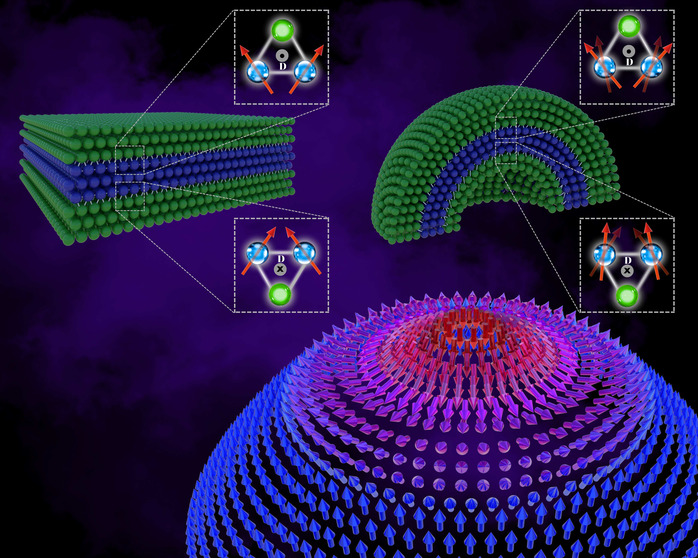

Skyrmions are topologically nontrivial magnetic structures that have drawn significant interest in condensed matter physics and materials science.^1–4^ Their topological charge provides stability against external disturbances.^5^ With their small size (on the nanometer scale) and low energy requirements for manipulation,^6,7^ they show promise for applications in data storage,^8–10^ logic gates,^11,12^ spintronics, and neuromorphic computing.^13,14^ As a result, research into stabilizing and controlling skyrmions is critical for future technological innovations.

Skyrmion stability arises from the interplay of magnetic energy terms, including magnetostatics,^15–17^ exchange interaction, perpendicular magnetic anisotropy (PMA),^18^ and Dzyaloshinskii–Moriya interaction (DMI).^19,20^ In thin films, DMI takes an interfacial form (iDMI), where a heavy metal (HM) mediates the canting of spins in a ferromagnetic material (FM).^7^

Nanometer-thick magnetic multilayers, composed of alternating HM and FM layers, offer fertile ground for exploring magnetism.^21^ These systems exhibit enhanced iDMI^22–26^ and other phenomena like PMA.^18,27–30^

Interest in curved systems such as spheres and cylinders is growing.^31–35^ Curvature can be used to design magnetic structures with unique functionalities, influencing spin configurations and generating new topological characteristics.^36^ Recent studies^37^ have shown that curvature in magnetic spherical half-shells stabilizes various textures, including vortex, monodomain, skyrmion, and skyrmionium states. Moreover, reviews and roadmaps can be found in refs 38–41 and references therein.

This work investigates how the geometry of curved nanomagnets (nanocaps) affects the magnetic anisotropy (*K*), magnetostatic energy, and iDMI, aiming to identify the optimal parameters for stabilizing skyrmions in zero fields. We first used density functional theory (DFT) to show that bending a surface induces symmetry breaking, enhancing iDMI. Micromagnetic simulations of curved nanomagnets were then conducted to find the best parameters for skyrmion stabilization. The magnetic nanocaps were fabricated using Co/Pt multilayers over polystyrene nanospheres. Characterization was performed using a vibrating sample magnetometer (VSM), magnetic force microscopy (MFM), and electron holography, combined with micromagnetic simulations, to assess the magnetic texture.

Although HM/FM interfaces are expected to exhibit iDMI, symmetric HM/FM/HM systems with flat interfaces typically show negligible iDMI due to cancelation of contributions from the top and bottom interfaces.^7,42–45^ However, some symmetry breaking, such as imperfections, can induce significant iDMI.^46^ In our case, the curvature of the nanocaps would cause a tunable breaking of symmetry, which is suitable for making a tailored iDMI, as shown in Figure 1a.

**Figure 1:**
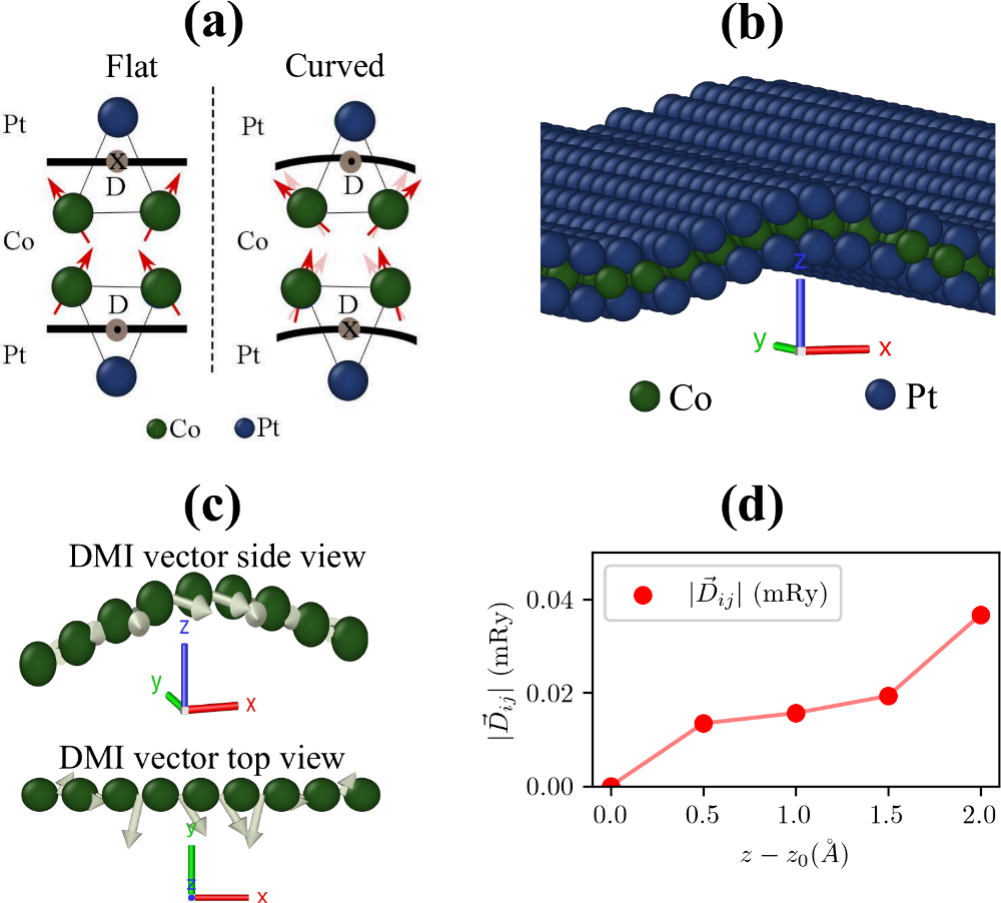
(a) Schematic illustration of spins and iDMI in flat and curved symmetric Pt/Co/Pt, showing the symmetry break induced by curvature. (b) Simulated curved PtCoPt surface via DFT, with blue spheres for Pt atoms and green spheres for Co atoms. The unit cell includes curvature along the *x*-axis and boundary conditions along the *y*-axis. (c) Side and top views of the calculated iDMI vector between nearest neighbor atoms, with arrows denoting direction and strength. iDMI increases near the top of the structure, where curvature is more pronounced. (d) iDMI amplitude as a function of curvature factor.

To demonstrate symmetry breaking and the emergence of iDMI, we performed atomic-level DFT calculations analyzing magnetic interactions as a function of curvature. The spin Hamiltonian parameters are given by

1 $$H=-\underset{\left\langle ij\right\rangle }{\sum }\left(J_{ij}{\mathrm{e⃗}}_{i}\cdot {\mathrm{e⃗}}_{j}+{\mathrm{D⃗}}_{ij}\cdot \left({\mathrm{e⃗}}_{i}\times {\mathrm{e⃗}}_{j}\right)\right)$$

where ⟨*ij*⟩ is the summation over the pairs to avoid double summation, *J*_*ij*_ is the Heisenberg exchange, and *D⃗*_*ij*_ is the iDMI vector. Magnetic parameter calculations are detailed in the Supporting Information (Section S1). While DFT cannot replicate experimental setups due to cost, it illustrates how atomic-scale curvature induces iDMI in Co/Pt systems, generalizable to the microscopic scale. A 33-atom primitive cell (11 Co and 22 Pt) was modeled with curvature along the *x*-axis and boundary conditions on the *y*-axis, as shown in Figure 1b.

If the surface is flat, the inversion symmetry remains intact, and the iDMI vector is null. However, the Heisenberg exchange is finite, with *J*_*ij*_^flat^ = 0.72 mRy, indicating ferromagnetism according to eq 1. Inducing curvature by shifting Co and Pt atoms along the *z*-axis (Figure 1b) breaks this symmetry, resulting in *J*_*ij*_^curved^ = 0.62 mRy and |*D⃗*_*ij*_| = 0.04 mRy. The iDMI vector predominantly lies in the *y* direction perpendicular to the bond between Co neighbors (Figure 1c, top).

In flat cubic surfaces (e.g., vacuum-Co-Pt) with symmetry breaking in the 001 direction, the iDMI vector aligns in-plane and perpendicular to the bond, consistent with Moryia rules.^47^ Curvature further breaks symmetry along the *x*- and *z*-axes, placing the iDMI vector in the *xy* plane, although its *y*-component dominates. At the edges, weaker curvature results in lower iDMI values compared to the top (Figure 1c, bottom).

In one dimension, the relation between *J*_*ij*_ and *D⃗ij* would produce spin spirals with periods modulated by iDMI strength. On a surface, this leads to skyrmions, whose radius depends on these interactions. Figure 1d shows how curvature, represented by atomic displacement in the *z*-axis (*z* – *z*_0_), affects iDMI. For flat surfaces (*z* – *z*_0_ = 0), iDMI is null. As curvature increases, iDMI strength rises, highlighting curvature’s significant role in modulating iDMI.

The curvature-driven emergence of nonzero iDMI influences the stabilization of complex magnetic textures like skyrmions.^31^ The nanocap diameter is linked to curvature (Supporting Information, Section S3.1) and determines iDMI strength, as shown in Figure 1d. Magnetostatic energy also depends on nanocap diameter,^16^ while Co thickness in the multilayer adjusts the magnetic anisotropy constant (*K*).^43^ By tuning parameters such as the curvature and FM thickness, the amplitude of these energy terms can be manipulated independently, making bent Co/Pt multilayers an archetypic system.

To identify suitable magnetic parameters for stabilizing skyrmions in nanocaps, we constructed a micromagnetic phase diagram for *K* and iDMI using Mumax3 ^48^ (details in Supporting Information, Section S2). Keeping the nanocap diameter constant at 500 nm to fix the magnetostatic energy, we varied the other parameters independently.

A magnetic film with strong perpendicular magnetic anisotropy (*K*_eff_) typically exhibits uniform magnetization perpendicular to the surface. However, when bent, the magnetization aligns perpendicular to the local surface, resulting in a radial anisotropy distribution for Co/Pt nanocaps with a large *K*_eff_. To model this, we defined a radial anisotropy in simulations, with the amplitude given by *K*_radial_. Additionally, the nanocaps exhibit a thickness gradient, being thicker at the top and thinner at the edges due to the fabrication method. Both features were included in the simulations, as shown in Figure 2a (top, side, and cross-section views).

**Figure 2:**
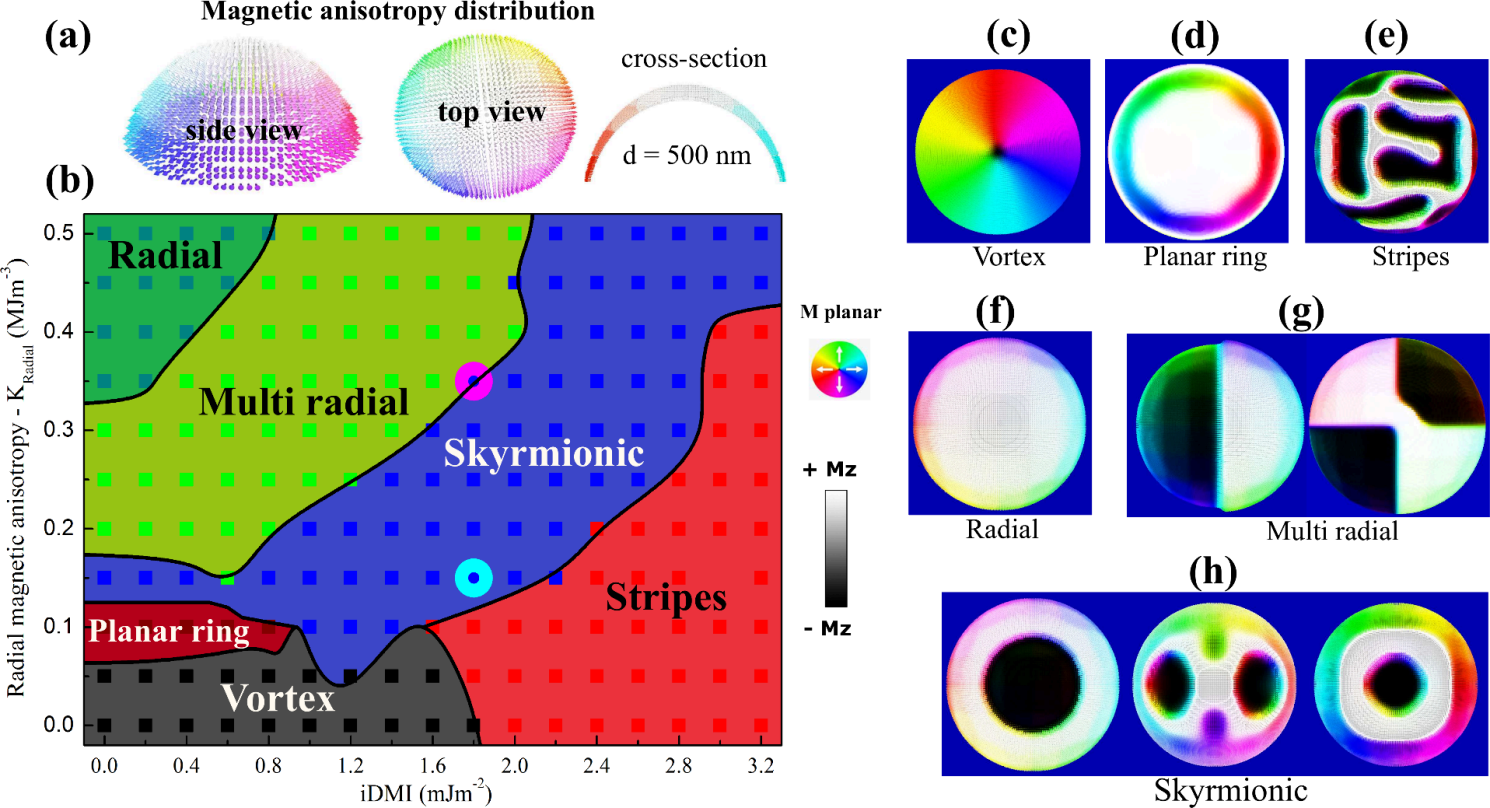
Zero magnetic field micromagnetic simulated domain patterns of a 500 nm diameter curved nanomagnet. (a) Geometry and magnetic anisotropy distribution of the simulated system. (b) Phase diagram for different *K*_radial_ and iDMI. (c) Simulated vortex state. (d) Planar ring state. (e) Stripes state. (f) Radial state. (g) Multiradial state. (h) Skyrmionic state. Images in (c–h) show the top view of the curved nanomagnet, with a diameter of 500 nm.

Simulations used the experimentally measured saturation magnetization of 550 kAm^–1^ ^43^ and *K*_radial_ values ranging from 0 MJm^–3^ (thick Co) to 0.5 MJm^–3^ (thin Co). The phase diagram of *K*_radial_ versus iDMI is shown in Figure 2b (details in Section S2 of the Supporting Information). Zero iDMI corresponds to perfectly flat Pt/Co/Pt interfaces, while increasing iDMI reflects enhanced nanocap curvature.

Figure 2b shows that micromagnetic simulations yield various magnetic ground states depending on *K*_radial_. For the low regime range, the vortex states (black region, Figure 2c) and the stripe domains (red region, Figure 2e) dominate. As *K*_radial_ increases, planar ring states (dark red region, Figure 2d) appear at low iDMI, while single and multiradial domains (light and dark green regions, Figures 2f and g) stabilize at higher *K*_radial_. At intermediate iDMI values, skyrmionic states (blue region, Figure 2h) stabilize, including Néel skyrmions, two Néel skyrmions, and skyrmionium.^49^ These findings enable the design of nanocap arrays for specific textures like skyrmions.

However, the *K*_eff_ obtained experimentally from the difference in hysteresis loop areas under in-plane and out-of-plane fields does not directly match the *K*_radial_ used in simulations. This discrepancy arises because radial anisotropy differs from uniaxial perpendicular anisotropy, and iDMI enhances the latter. To define properties for desired textures, we simulated hysteresis loops in regions where these textures are ground states. One loop was simulated with an out-of-plane field (*M*_oop_) and the other with an in-plane field (*M*_ip_), and *K*_eff_ was calculated as the difference between loop areas (Figure 3), i.e.,

2 $$K_{eff}=\left(\int M_{oop}dH-\int M_{ip}dH\right)/2$$

This methodology correlates *K*_eff_, *K*_radial_, and iDMI, as shown by comparing Figures 3 and 2b.

**Figure 3:**
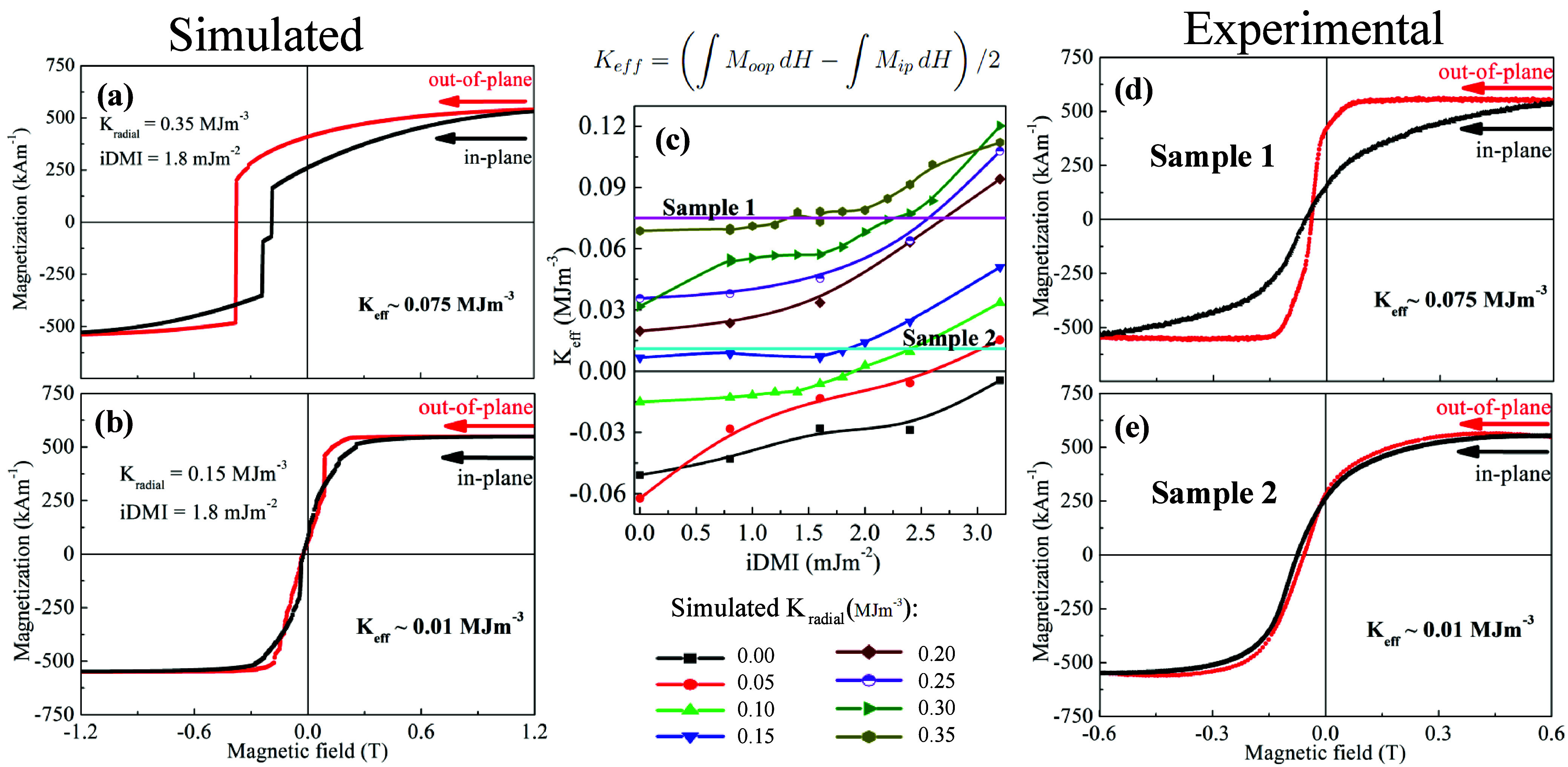
Micromagnetic simulation and experimental magnetization curves with in-plane and out-of-plane magnetic field. (a) For simulated *K*_radial_ = 0.35 MJ m^–3^ and *D* = 1.8 mJ m^–2^ and (b) for *K*_radial_ = 0.15 MJ m^–3^ and *D* = 1.8 mJ m^–2^. These curves correspond to the points highlighted in the phase diagram: (a) pink point and (b) cyan point. (c) Calculated effective magnetic anisotropy constant (*K*_eff_) from hysteresis loops as a function of iDMI for different *K*_radial_ values. *K*_eff_ corresponds to the difference in area between the out-of-plane and in-plane curves, as described by the equation highlighted (eq 2). (d) Experimental magnetization curves measured in a VersaLab-Quantum Design VSM at room temperature for *K*_eff_ ≈ 0.075 MJ m^–3^ (d) and *K*_eff_ ≈ 0.01 MJ m^–3^ (e) on a PS 500 nm.

The next challenge is to fabricate nanocap arrays with experimentally measured *K*_eff_ values closely matching simulation results. Comparing *K*_eff_ values from simulations (Figure 3) and experimental hysteresis curves determines the iDMI range of the real sample.

According to the phase diagram, skyrmionic states are stable for *K*_radial_ = 0.1 to 0.5 MJ m^–3^ and iDMI = 0 to 3.2 mJ m^–2^. These *K*_radial_ values correspond to *K*_eff_ between −0.06 and 0.12 MJ m^–3^, as shown in Figure 3c, where the calculated values for *K*_eff_ for different values of *K*_radial_ and iDMI are presented. It should be noted that these values for *K*_eff_ can be achieved experimentally. *K*_radial_ ≈ 0.15 MJ m^–3^ offers optimal conditions for skyrmions due to its broad iDMI range, including zero iDMI. Samples with *K*_eff_ between 0.01 and 0.1 MJ m^–3^ are suitable for stabilizing skyrmions, provided the iDMI is appropriate. Thus, nanocap arrays with Co/Pt multilayers of varying Co thicknesses were fabricated to target these values.

Nanocap arrays with thicknesses of *t*_*n*_ (Figure S6) were fabricated using [Pt­(1 nm)/Co­(*t*)/Pt­(1 nm)]_10_, where *t* is Co thickness. Hysteresis loops for in-plane and out-of-plane fields were measured to determine *K*_eff_, using methods similar to those used in simulations. The hysteresis curves for [Pt­(1 nm)/Co­(2 nm)/Pt­(1 nm)]_10_ are shown in Figure 3d.

Experimentally, *K*_eff_ ranged from 0.075 MJ m^–3^ (sample 1, pink line in Figure 3c) to 0.01 MJ m^–3^ (sample 2, cyan line). This range aligns with skyrmion stability.

This methodology also estimates the iDMI values. For sample 1 with *K*_eff_ ≈ 0. 075 MJ m^–3^, the iDMI is 1.6 ± 0.4 mJ m^–2^, while for sample 2 with *K*_eff_ ≈ 0. 01 MJ m^–3^, the iDMI is 1.8 ± 0.4 mJ m^–2^.

After identifying the sample with suitable magnetic properties (*M*_s_ and *K*_eff_) for stabilizing skyrmionic phases, we examined, in the remanent state, its magnetic textures using MFM (details in Supporting Information, Section S3.2). Figure 4a shows the magnetic image of the CoPt multilayer grown on a 500 nm nanosphere array at zero field (sample 1 with *K*_eff_ ≈ 0.075 MJ m^–3^, Figure 3d). Predominantly dark domains suggest radial orientations consistent with simulations, while bright spots (Figure 4b) surrounded by dark contrast indicate skyrmion-like structures.^37,49^ The topographic image (Figure S8c) confirms that these contrasts are not topographical.

**Figure 4:**
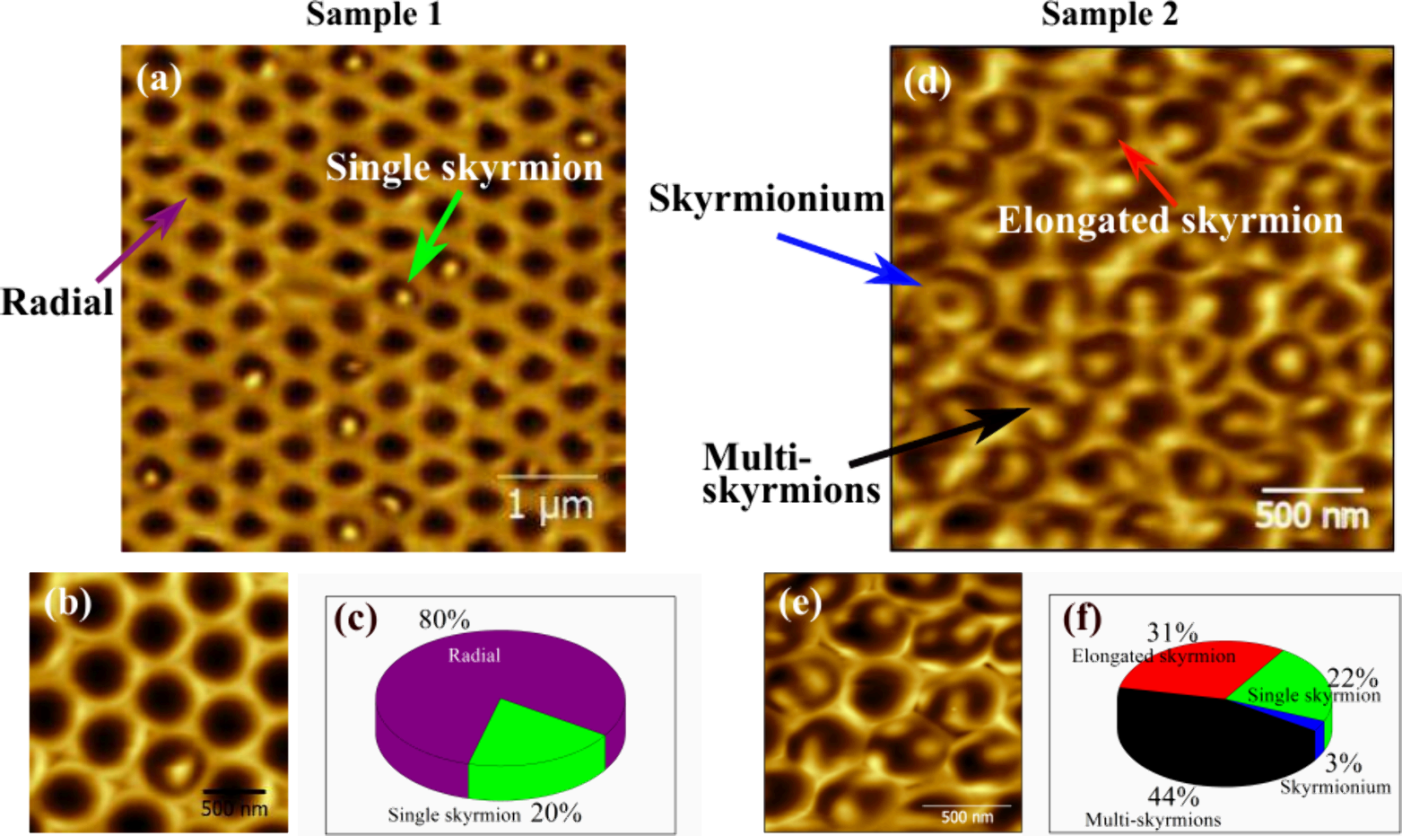
(a) Punctual magnetic skyrmions on curved nanocaps at zero field for sample 1 (*K*_eff_ ≈ 0.075 MJ m^–3^), measured by magnetic force microscopy (MFM) in the remanent magnetization state. (b) Zoom-in on a 2 × 2 μm^2^ region, highlighting a circular domain on the nanocap. (c) A graph presenting the statistics on the prevalence of the observed texture shows that, in this sample, only radial domains and single skyrmions are observed. (d) MFM image of sample 2 (*K*_eff_ ≈ 0.01 MJ m^–3^), from where different skyrmionics states are observed, namely, single-skyrmion, skyrmionium, elongated skyrmion, and multiskyrmions. (e) Zoom-in on a 2 × 2 μm region, highlighting a circular domain on the nanocap. (f) A graph presenting the statistics on the prevalence of the observed skyrmionics identified.

We extended the analysis to a larger area (≈ 1000 nanocaps) for statistical evaluation (Figure S9). Radial monodomain states were predominant, with skyrmions found in ≈20% of nanocaps. This statistic is presented in Figure 4c. The average skyrmion size, determined from the full-width at half-maximum (fwhm) of the profile, was ≈140 nm (Figure S8d).

The ≈20% prevalence of skyrmions in the *K*_eff_ ≈ 0. 075 MJ m^–3^ sample is attributed to its iDMI value being near a phase boundary. Here, the energies of the radial monodomain and skyrmion states are nearly degenerate, forming a quasi-stable system. At the boundary (iDMI ≈ 1.8 mJ m^–2^), the energy difference is ≈0.2% (Figure S5). This allows the estimated iDMI to be refined to 1.8 ± 0.2 mJ m^–2^, as indicated by the pink circle in the phase diagram (Figure 2). Due to curvature-induced symmetry breaking, other samples fabricated on 500 nm polystyrene nanospheres are expected to exhibit similar iDMI values.

An attentive examination of the hysteresis loop of sample 1 (Figure 3b) reveals that the remanent magnetization reaches approximately 80% saturation magnetization. We acknowledge the strong correlation between the prevalence of skyrmions (20%) and the remanent magnetization (80%), which may suggest a direct association between these effects. However, we argue that the remanence in this system is more complex. In particular, the magnetic anisotropy is not strictly perpendicular but rather exhibits a radial component. Consequently, even in the absence of skyrmions, the system would still display a remanent magnetization lower than the saturation magnetization. Therefore, any direct association between these observations would be premature.

Reevaluation of the phase diagram indicates that lowering *K*_eff_ could enhance skyrmion prevalence. Therefore, we examined sample 2 with *K*_eff_ ≈ 0. 01 MJ m^–3^ (Figure 3e) and *K*_radial_ = 0.15 MJm^–3^ (Figure 3b). This sample, maintaining a nanocap diameter of 500 nm, is expected to exhibit an iDMI of 1.8 ± 0.2 mJ m^–2^. Its location in the phase diagram (cyan point) affirms its capability to stabilize skyrmionic states. This is corroborated by MFM measurements, as presented in Figure 4 (for more detail, see Supporting Information, Figures S10 and S11). As observed, the magnetic structures included single and multiskyrmions, elongated skyrmions, and skymioniums. Each texture’s prevalence is presented in the graph in Figure 4f. As observed, the most abundant skyrmionic states are multi- and elongated skyrmions. However, it is interesting to note that approximately 20% of the sample consists of single skyrmions, while 3% display a texture that we could identify as skyrmionium. Although we need a deeper analysis to confirm this, it is worth noting that skyrmionium is an exotic texture that is hardly observed experimentally under ambient conditions. These findings align with simulations indicating that skyrmionic textures represent the lowest energy states within this range of the diagram (Figure 2h). No radial or monodomain states were detected, demonstrating that the sample is significantly distanced from the phase boundaries.

We recognize that MFM may not be the most dependable method for confirming skyrmions or intricate textures. Hence, to validate our MFM findings with improved spatial resolution, we conducted electron holography on the chosen samples.

The electron holography and micromagnetic simulations in Figure 5 confirm the presence of a single skyrmion state and two skyrmion states in nanocaps. The mean inner potential phase image (Figure 5a) shows the nanocap morphology, and the schematic (Figure 5b) indicates the skyrmion’s position. The curvature provides the tilt angle necessary for holography to detect the in-plane magnetic component, ϕ_*m*_, of the skyrmion. The ϕ_*m*_ phase image (Figure 5c) and magnetic induction map (Figure 5d) show contrast consistent with Néel skyrmions in thin films.^50–52^ Micromagnetic simulations of a single Néel skyrmion (Figure 5e) tilted to 20° (Figure 5f) produce a projected ϕ_*m*_ phase image (Figure 5g) and a magnetic induction map (Figure 5h), agreeing with experimental results. Further comparison with idealized ϕ_*m*_ values validates these observations (Supporting Information, Section S3.3.2).

**Figure 5:**
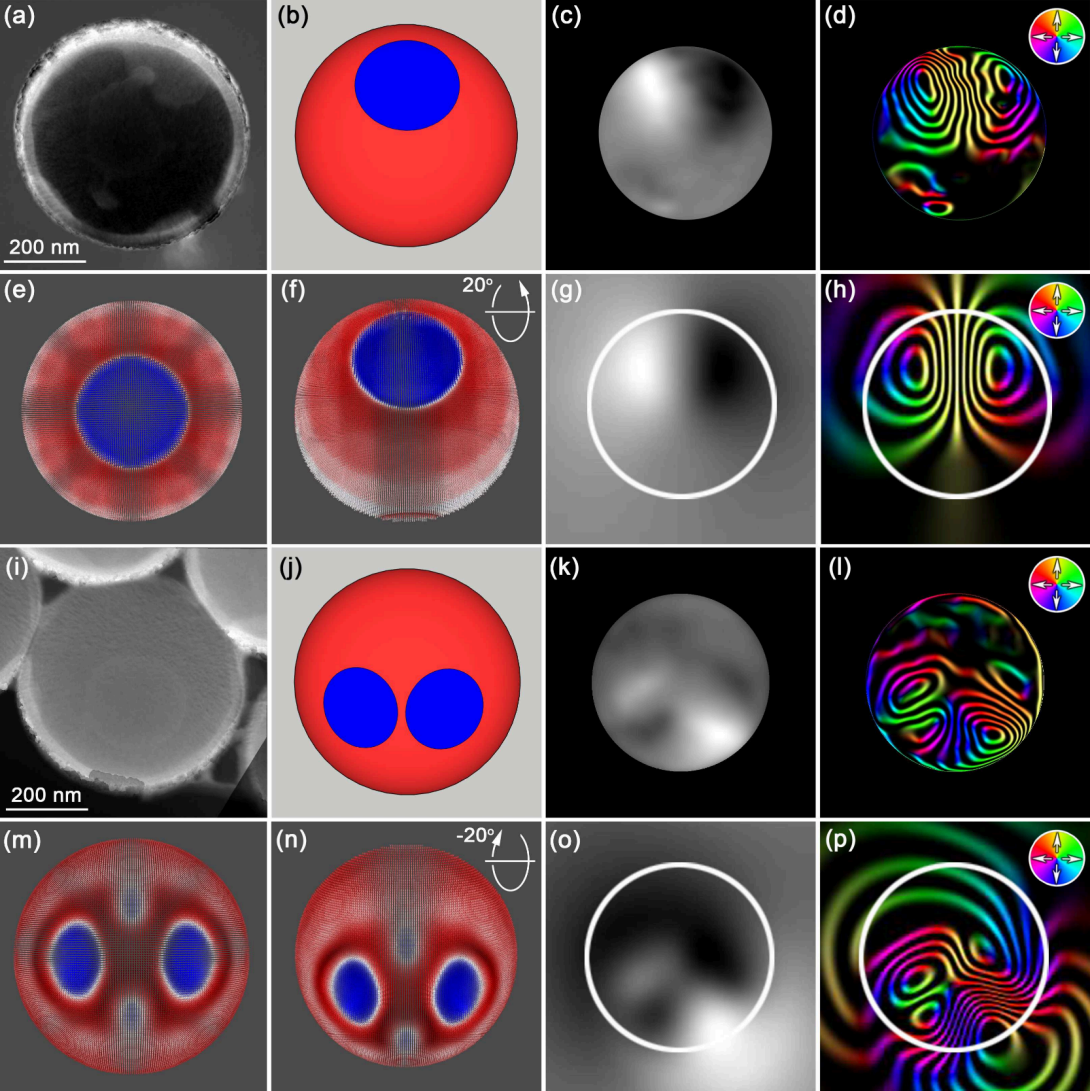
Electron holography and micromagnetic simulations of magnetic induction within nanocaps. (a) Mean inner potential phase image of a 500 nm nanocap. (b) Schematic showing a single skyrmion on the curved edge. (c) Magnetic phase image. (d) Magnetic induction map of the skyrmion (cosine amplified × 40) with magnetization direction indicated by the color wheel (inset). (e, f) Simulations of the single skyrmion at (e) 0° and (f) 20° tilt. (g, h) Projected magnetic phase image and induction map at 20° tilt. (i) Mean inner potential phase image of a 500 nm nanocap. (j) Schematic showing two skyrmions on the curved edge. (k) Magnetic phase image; (l) magnetic induction map of the two skyrmions (cosine amplified × 50) with magnetization direction indicated by the color wheel (inset). (m, n) Simulations of two Néel skyrmions at (m) 0° and (n) −20° tilt. (o, p) Projected magnetic phase image and induction map of two Néel skyrmions at 20° tilt.

Figure 5i shows the morphology of a nanocap with two skyrmions, and the schematic (Figure 5j) indicates their positions. The ϕ_*m*_ phase image (Figure 5k) and magnetic induction map (Figure 5l) confirm the presence of two Néel skyrmions. Simulations of a nanocap with two skyrmions (Figure 5m), tilted to −20° (Figure 5n), produce a projected ϕ_*m*_ phase image (Figure 5o) and a magnetic induction map (Figure 5p), again consistent with experimental results. Thus, the electron holography results and simulated phase images provide unambiguous evidence of single and two Néel skyrmion states in the nanocaps.

The interplay between topographical features and skyrmion formation holds significance for designing and optimizing magnetic materials and nanodevices. The incorporation of skyrmion stabilization in curved nanocaps has the potential to be applied in memory devices.^8,10^ Continued investigations into their stability, interaction, motion dynamics, and smaller sizes in curved systems will undoubtedly enhance our comprehension of their characteristics, potentially paving the way for innovative applications in the realms of spintronics and skyrmionics.

In summary, we demonstrated that curved Co/Pt multilayers (nanocaps) are an archetypical system for stabilizing skyrmions by manipulating key energies: magnetostatic, exchange interaction, perpendicular anisotropy, and interfacial Dzyaloshinskii–Moriya interaction. These energies can be tuned by modifying sample geometry, such as the curvature and FM thickness. DFT calculations showed that bending a magnetic surface breaks symmetry, generating significant iDMI. Micromagnetic simulations identified the critical parameters for skyrmion formation without external stimuli, guiding an effective manufacturing process. Experimentally, we fabricated curved magnetic multilayers and confirmed the stabilization of skyrmions in Co/Pt nanocap arrays without magnetic fields or other external stimuli. This research highlights the potential for localized skyrmion stabilization, offering promising applications for skyrmionic-based devices.

## Supplementary Material



## Notes

Detailed descriptions of the micromagnetic simulation parameters, additional MFM images of skyrmionic structures, further hysteresis curve analyses, and complementary electron holography data (PDF)
