# Supplementary material for: Curved Nanomagnets: An Archetype for the Skyrmionic States at Ambient Conditions
Source: Nano Lett. 2025 Apr 10;25(22):8901–8. doi: 10.1021/acs.nanolett.5c00773 (PMC12142663; doi:10.1021/acs.nanolett.5c00773)
Supplement: Supplementary file 1 [file nl5c00773_si_001.pdf]

Supporting Information

Curved Nanomagnets: An Archetype for the Skyrmionic States at Ambient Conditions

Danian A. Dugato,*,† Wesley B.F. Jalil,† Ramon Cardias,‡ Marcelo Albuquerque,‡ Marcio Costa,‡ Trevor P. Almeida,¶ Kayla Fallon,¶ András Kovács,§ Stephen McVitie,¶ Rafal E. Dunin-Borkowski,§ and Flávio Garcia*,†

†*Centro Brasileiro de Pesquisas Físicas (CBPF), Rua Dr Xavier Sigaud 150, Urca, 22290-180, Rio de Janeiro-RJ, Brazil*

‡*Instituto de Física, Universidade Federal Fluminense, 24210-346 Niterói RJ, Brazil*

¶*SUPA, School of Physics and Astronomy, University of Glasgow, Glasgow G12 8QQ, United Kingdom*

§*Ernst Ruska-Centre for Microscopy and Spectroscopy with Electrons and Peter Grünberg Institute, Forschungszentrum Jülich, 52425 Jülich, Germany*

E-mail: dgt.danian@gmail.com; flavio.cbpf@gmail.com

S1 - Density Functional Theory Simulations

We performed Density Functional Calculations (DFT)[1,2] using the plane wave-based code Quantum Espresso.[3] The interaction among the electrons was included via the generalized gradient approximation (GGA).[4] The ionic cores effects were treated using projected augmented wave (PAW) potentials.[5] A 52 Ry wavefunctions energy cutoff was used. The charge density cutoff is ten times larger. To model the experimental setup a trilayer of

Pt/Co/Pt was constructed. The unit cell is oriented along the 001 direction of the Pt-fcc crystal. We also kept the Pt lattice parameter, allowing the trilayer structure to fully relax until the forcer was smaller than 0.01 eV/Å. Since we use periodic boundary conditions to avoid spurious interactions (between the images), a distance of 15 Å was used along the trilayer perpendicular direction.

In order to investigate the exchange and Dzyaloshinskii-Moriya interaction (DMI) changes due to the trilayer curvature, we build a large supercell along the x-direction as shown in Figure S1(a). The curvature was generated by a sinodal modulation along the x-direction, where the displacement is Figure S1(b). The curved structural parameters were kept fixed. Our analysis of the exchange Dzyaloshinskii-Moriya interactions is focused on the Co central atom.

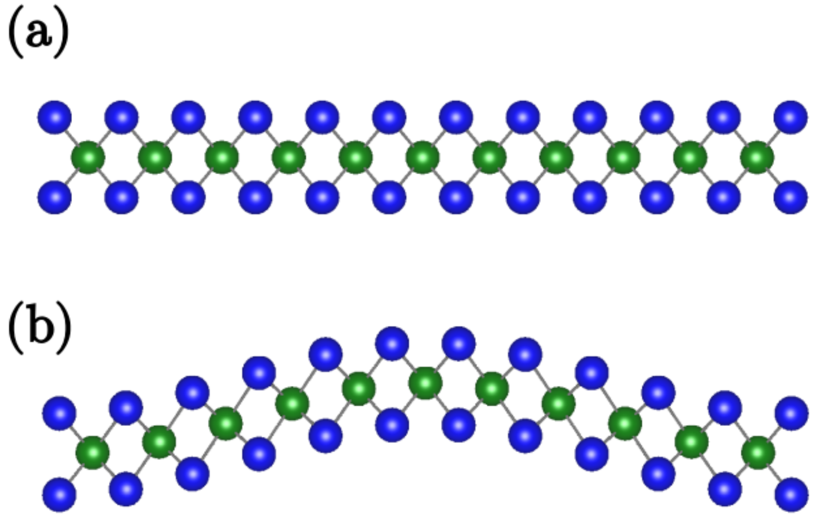

Figure S1: Side view of the Pt/Co/Pt trilayer supercell along the x-direction. (a) flat and (b) curvature induced by a sinodal modulation. The Pt and Co atoms are represented by blue and green spheres, respectively.

S1.1 - Exchange and Dzyaloshinskii-Moriya interaction calculations

To calculate the exchange and Dzyaloshinskii-Moriya interactions, we used the so-called Liechtenstein-Katsnelson-Antropov-Gubanov (LKAG) method.[6] The LKAG method uses

the magnetic force theorem by mapping the electronic Hamiltonian onto a classical spin Hamiltonian, whose parameters are calculated using Equations S1 and S2.

$$J_{ij} = \frac{1}{4\pi}\Im \int \mathrm{Tr}(\Delta_i\, G_{ij}^{\uparrow\uparrow}\, \Delta_j G_{ji}^{\downarrow\downarrow})\mathrm{d}\varepsilon\,, \tag{S1}$$

$$\vec{D}_{ij} = \frac{1}{4\pi}\Re \int \mathrm{Tr}_L \left(\Delta_i\, G_{ij}^{0}\, \Delta_j\, \vec{G}_{ji} + \Delta_j\, G_{ji}^{0}\, \Delta_i\, \vec{G}_{ij}\right) \mathrm{d}\varepsilon\,, \tag{S2}$$

where, $\Delta_i = \left(\mathcal{H}_{ii}^{\uparrow\uparrow} - \mathcal{H}_{ii}^{\downarrow\downarrow}\right)$ is the on-site exchange splitting and G_{ij} is the inter-site Green's function which, in our case, is calculated by using the Lanczos recursion method.

The LKAG equations are written in terms of the site projected Green's function; therefore, a delocalized basis, such as plane waves (PW), is not suitable. So we need to perform a basis change. To accomplish this task, we employed the pseudo atomic orbital (PAO) projection method[7–9] as implemented in the code PAOFLOW.[10,11] The PAO method involves projecting the Kohn-Sham orbitals, which consist of several thousand plane waves (PWs), onto the pseudo atomic orbitals, naturally built in the pseudopotential used in the DFT calculation. The PAO method will produce a real space-effective Hamiltonian (tight-binding-like) reproducing the DFT band structure. Our PAO Hamiltonians were constructed using *spd* orbitals for Co and Pt atoms. The atomic spin-orbit coupling (SOC) is introduced in the PAO Hamiltonian via an effective approximation. The resulting effective Hamiltonian is given by,

$$H_{PAO} = \sum_{ij}\sum_{\mu\nu}\sum_{\sigma} t_{ij}^{\mu\nu} c_{i\mu\sigma}^{\dagger} c_{j\nu\sigma} + \sum_{i}\sum_{\mu\nu}\sum_{\sigma\sigma'} \xi_i^{\mu\nu} \langle i\mu\sigma|\mathbf{L}\cdot\mathbf{S}|i\nu\sigma'\rangle c_{i\mu\sigma}^{\dagger} c_{i\nu\sigma'}, \tag{S3}$$

where the operator $c_{i\mu\sigma}^{\dagger}(c_{i\mu\sigma})$ creates (annihilates) an electron with spin projection σ at the atomic site i and orbital μ. Here, the Latin letters label atomic sites, while the Greek ones (except for σ) label the relevant atomic orbitals and the hopping matrix elements are given by $t_{ij}^{\mu\nu}$. The $\mathbf{L}$ and $\mathbf{S}$ are the orbital and spin angular momentum operators, respectively.

The SOC strength denoted as $\xi_i^{\mu\nu}$, is derived from fitting to a fully relativistic DFT calculation. The fitting process results in values of 0.09 eV for Co and 0.55 eV for Pt SOC strength. This methodology has been successfully used on other systems see References.[12,13] The Figure S2 presents the fully relativistic band structure for the DFT (a) and the PAO Hamiltonian ad-hoc SOC in (b). The agreement between the two band structures is excellent.

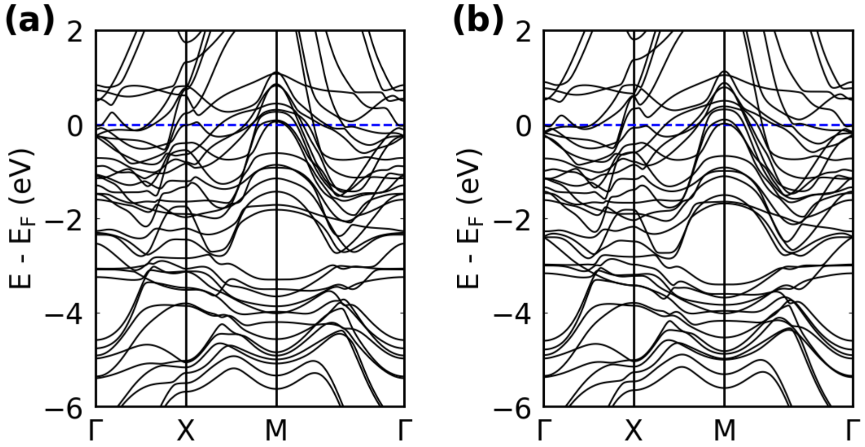

Figure S2: Pt/Co/Pt trilayer bulk band structure for (a) Fully relativistic DFT calculation and (b) Ad-hoc spin-orbit couplin (SOC) in the PAO Hamiltonian. We used values of 0.09 eV and 0.55 eV for the SOC strength of Co and Pt, respectively.

S2 - Micromagnetic simulation details

We conducted micromagnetic simulations using Mumax3, a package GPU accelerated to solve the Landau-Lifshitz-Gilbert (LLG) equation.[14] The simulations were carried out within a spherical semi-shell with a given diameter (500 nm) and a thickness gradient, ranging from a thicker film at the top to zero thickness at the nanocap edge, to mimic an actual nanocap. We adopted this profile for simulation because we know, based on transmission electron microscopy measurements in previous work,[15] that it corresponds to the profile of a multilayer deposited on a nanosphere.

This simulated nanocap profile resembles that presented in our previous work.[16]In the model, this is achieved by interposing two ellipses. Furthermore, our model was developed

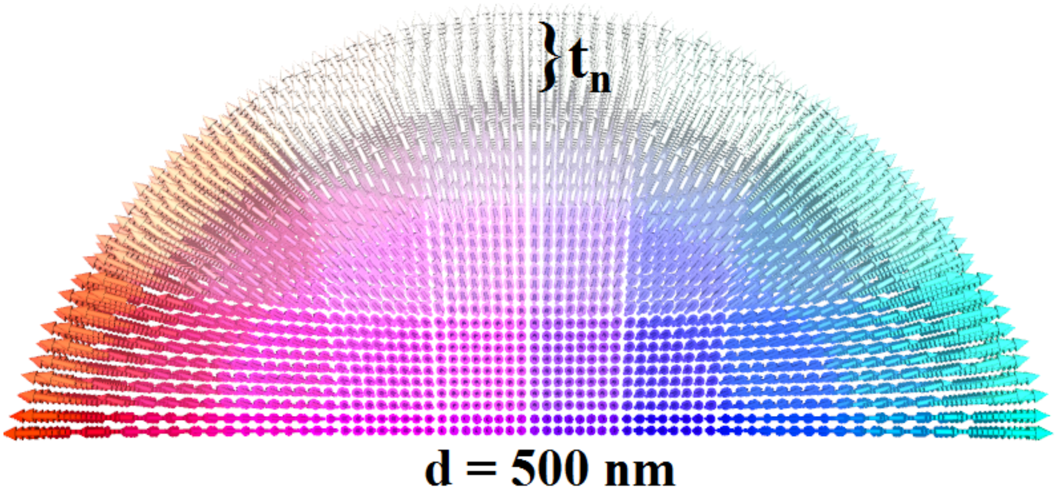

Figure S3: Cross section of the simulated nanocaps. The vectors indicate the orientation of the magnetic anistropy ($K_{\rm radial}$). We draw attention to the thickness gradient also present. The nominal thickness corresponds to the thickness of the top of the nanocap ($t_{\rm n}$).

with a radial distribution of magnetic anisotropy, to better represent the real (experimental) case (Figure S3) . The magnetic parameters used were magnetization of saturation of 550 kAm^{-1} (based on experimental measurements), the standard Co exchange stiffness (A = 12×10^{-12} Jm^{-1}), and damping $\alpha = 0.3$. The parameters of magnetic anisotropy ($K_{\rm radial}$) and the Dzyaloshinskii-Moriya interaction (DMI) were varied, as shown in the phase diagram of Figure 2 of the main text. We discretized the computational domain into cells measuring 3 x 3 x 3 nm^3 to ensure precision.

We initiate the simulation with nine distinct magnetic states: UniformZ, UniformX, Néel skyrmion, radial, 2 domains, 2 Néel skyrmions , vortex bimeron, 2 vortices, vortex , anti-vortex, skyrmionium, bimeron, Bloch skyrmion, skyrmion-2domain states. The initial states are presented in Figure S4. Subsequently, we allow the system to relax to achieve a minimum energy state.[16–18] The final magnetic configuration exhibiting the lowest energy level (exemple Fig. S5) represents the ground state presented in the phase diagram (Figure 2 - main text).

As mentioned in the main text, the anisotropy constant implemented in the phase diagram ($K_{\rm radial}$) does not correspond to the experimental anisotropy constant ($K_{\rm eff}$). In this way, to

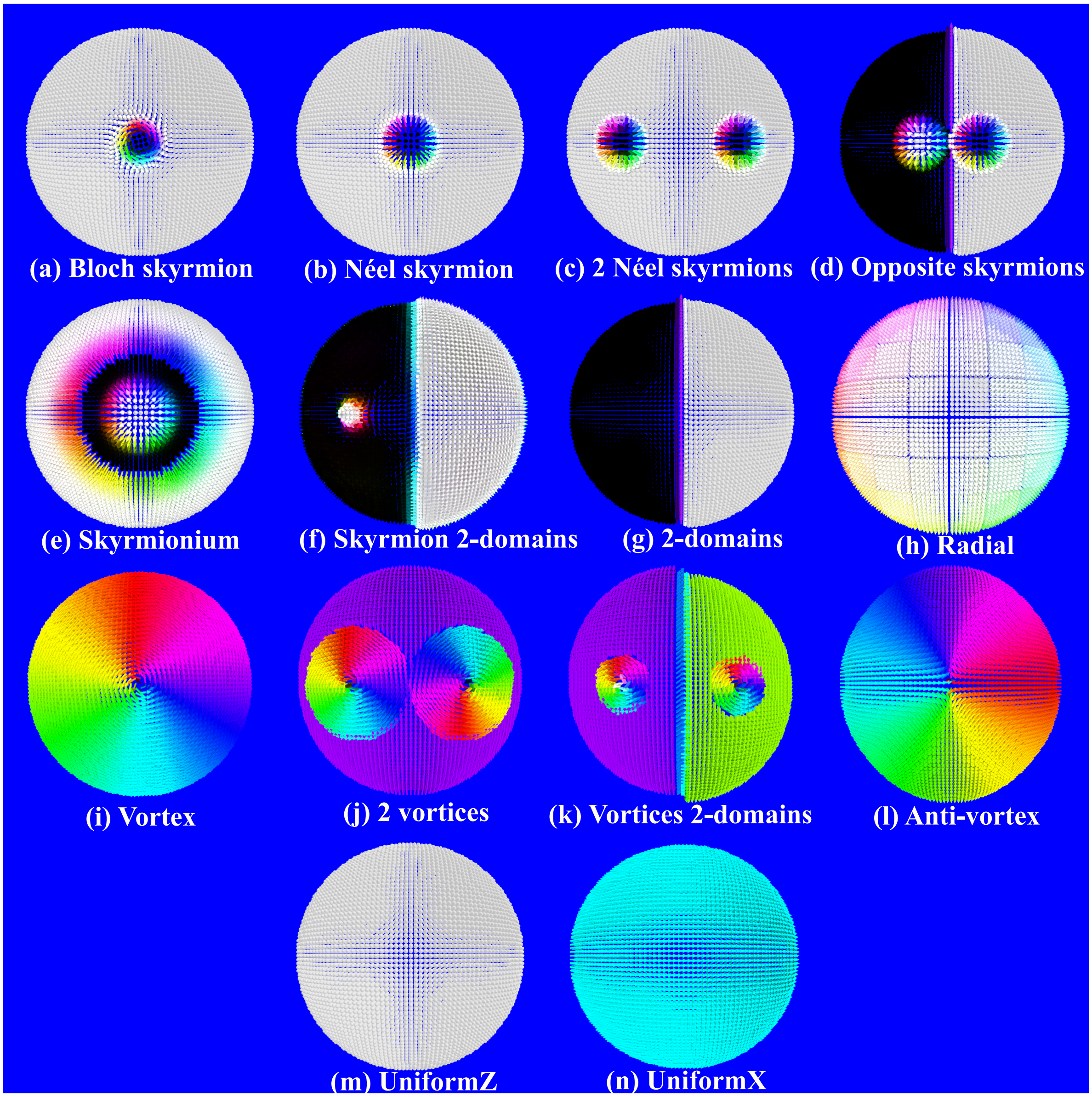

Figure S4: Initial states used in the micromagnetic simulation. (a) Bloch skyrmion, (b) Néel skyrmions, (c) 2 Néel skyrmions, (d) Opposite skyrmions, (e) Skyrmionium, (f) skyrmion 2-domain, (g) 2-domain, (h) Radial, (i) Vortex, (j) 2 vortices, (k) Vortices 2-domain, (l) Anti-vortex, (m) UniformZ, and (n) UniformX.

find where we are in the phase diagram with our experimental samples, we calculate the K_{eff} based on simulated hysteresis loops. Through the in-plane and out-of-plane magnetization curves, we obtain the K_{eff} taking the difference in area between the two magnetization curves. We calculate the area by integrating the area under the decreasing magnetic field curve for perpendicular and planar magnetic fields.

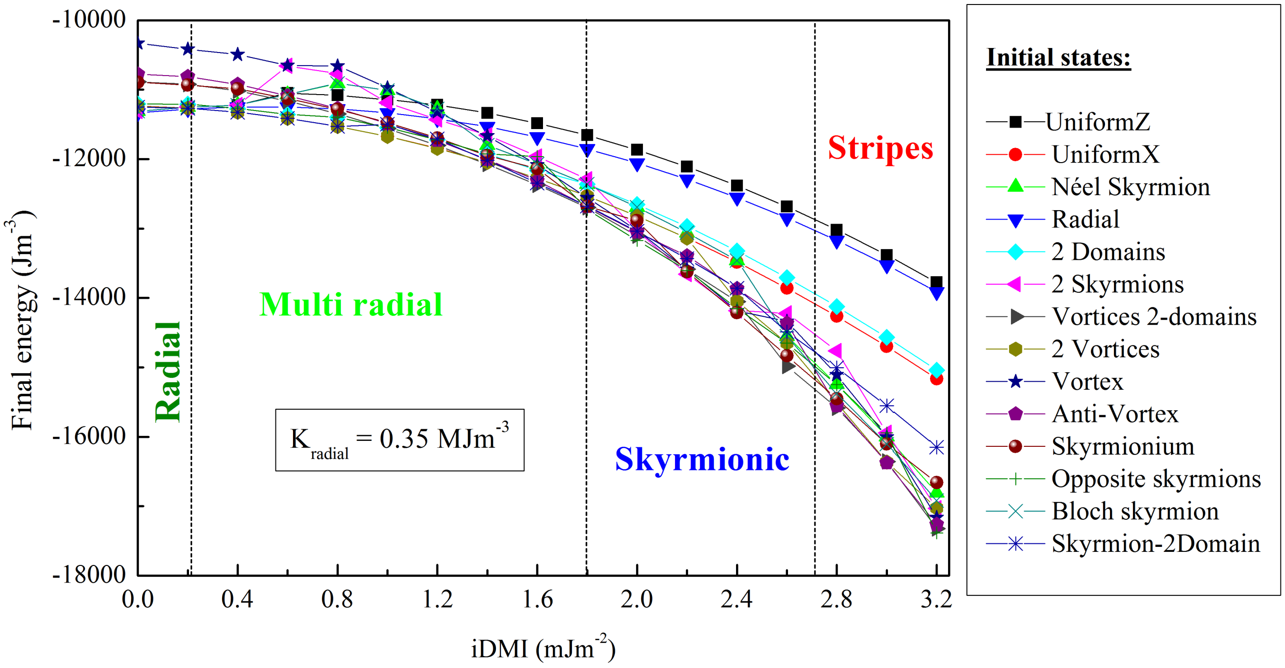

Figure S5: Final energy for difffent initial state as a function of iDMI for simulated $K_{\mathrm{radial}} = 0.35$ MJm^{-3}. These curves served as the basis for choosing the final magnetic state for each configuration presented in the phase diagram. The final magnetic state presented by the lowest energy condition is the ground state.

S3 - Experimental details

S3.1 - Sample fabrication

We created a polystyrene sphere (PS) template using colloidal lithography to investigate skyrmion nucleation in a curved multilayer. We used commercial 500-nm PS manufactured by Sigma-Aldrich. The methodology to produce the PS template was similar to that presented in our previous work.[16] Figure S6a illustrates the curved template manufacturing process. Initially, the PS is organized by a spin coater, and subsequently, the multilayer is grown. In Figure S6b, the profile of the nanocap resulting from the manufacturing method is depicted. The nanosphere is deposited only in the upper half and exhibits a gradient in the thickness of the multilayer. The nominal thickness of the multilayer (t_{n}) represents the

thickness at the highest point of the nanocap. This profile corresponds to a simulated profile by micromagnetic simulation.

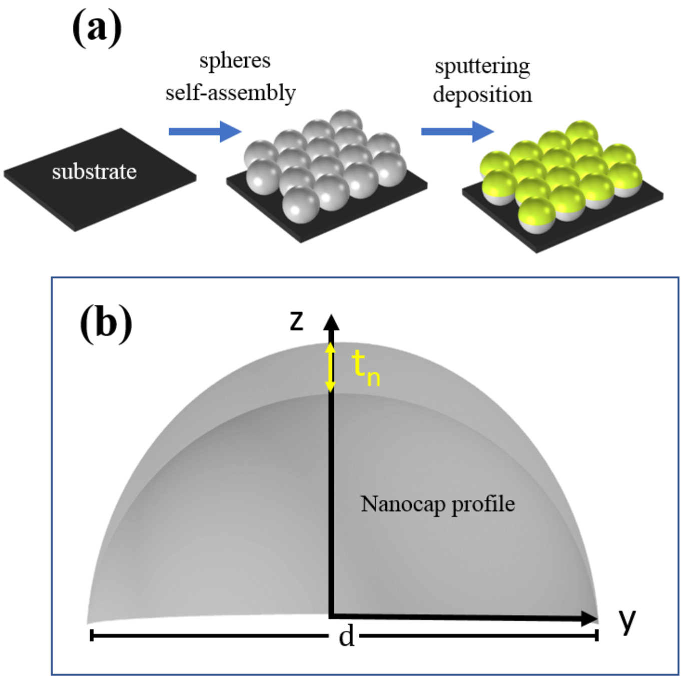

Figure S6: (a) Illustration of the manufacturing process of the PS template using colloidal lithography. (b) Profile of the nanomagnet.

To evaluate the organization of the nanospheres and their monolayer formation, we used the scanning electron microscopy (SEM) technique, as shown in Figure S7. We can observe an organized hexagonal packing of the PS template.

We grew a multilayer structure over this PS template using magnetron sputtering. The structure was comprised of $t_{\rm n}$ = [Pt (1 nm)/Co (t)/Pt (1 nm)] x10, with t ranging from 0.8 to 2.0 nm. This helped to achieve the required magnetic anisotropy for skyrmion stabilization.

S3.2 - Magnetic characterization

Magnetic parameters, such as saturation magnetization ($M_{\rm s}$) and anisotropy field ($H_{\rm k}$), were extracted from magnetic hysteresis curves measured in a VersaLab-Quantum Design vibrating sample magnetometer (VSM) at room temperature, yielding the perpendicular anisotropy constant value ($K_{\rm eff}$).

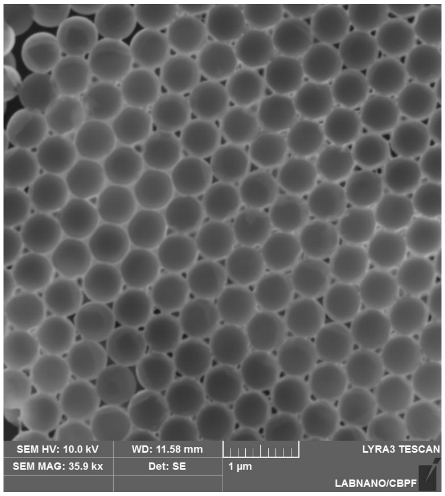

Figure S7: Scanning electron microscopy of 500 nm polystyrene spheres.

Atomic and magnetic force microscopy (AFM/MFM) techniques were used to obtain the topographical images and magnetic domain patterns of the as-grown nanocaps. Budget Sensors recorded the imaging using a Brucker microscope operating in tapping mode with Multi75-G MFM tips. In addition, the images were captured in a magnetic remanent state, under ambient conditions, without any external stimulus, at zero fields, with the probe positioned at a height of approximately 60 nm.

After identifying the sample with suitable magnetic properties (M_s and K_{eff}) for stabilizing skyrmionic phases, such as discussed on the mais text, we examined, in remanent state, its magnetic textures using MFM. Figure S8a shows the magnetic image of the CoPt multilayer grown on a 500 nm nanosphere array at zero field (Sample 1 with $K_{eff} \approx 0.075$ MJm^{-3}). Predominantly dark domains suggest radial orientations consistent with simulations, while bright spots (Figure S8b) surrounded by dark contrast indicate skyrmion-like structures. The magnetic profile (Figure S8d) aligns with skyrmion models. The topographic image (Figure S8c) confirms these contrasts are not topographical. A 3D projection of the nanocaps highlights how curvature stabilizes skyrmions (Figure S8e).

In Figure S9, we present the MFM image of this sample, with $K = 0.075$ MJm^{-3} (Sample 1), scanned over a large area (10 x 10 μm^2) to do the statistic about the skyrmion prevalence. Due to the large scanning area, some definition was lost, but it is still possible to observe a

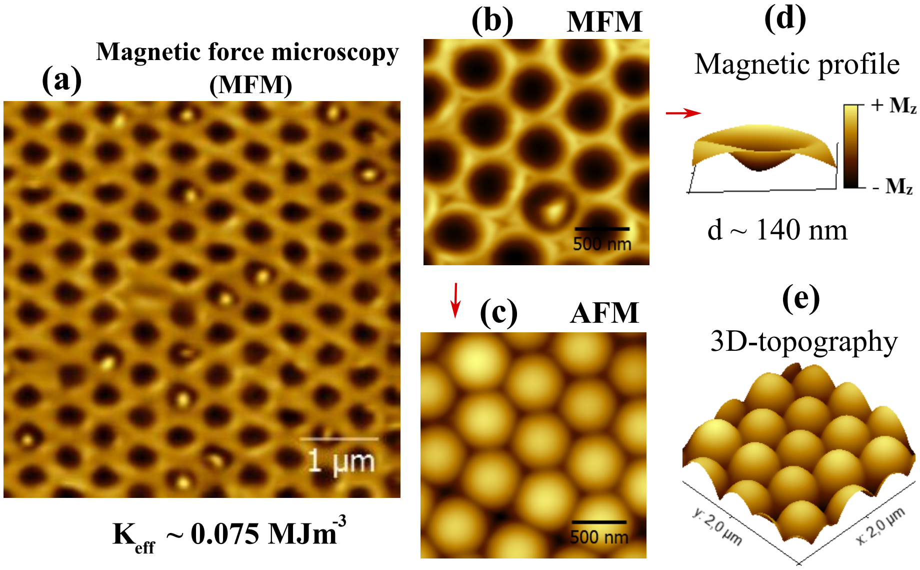

Figure S8: (a) Punctual magnetic skyrmions on curved nanocaps at zero field for the K_{eff} $\approx$ 0.075 MJm^{-3} sample (Sample 1), measured by magnetic force microscopy (MFM). (b) Zoom on a 2 x 2 µm region, highlighting a circular domain on the nanocap. (c) Topography synchronized with the previous magnetic image. (d) Profile traced in the magnetic image showing a distinct magnetic domain. (e) 3D projection of the topography, emphasizing the curvature of the system.

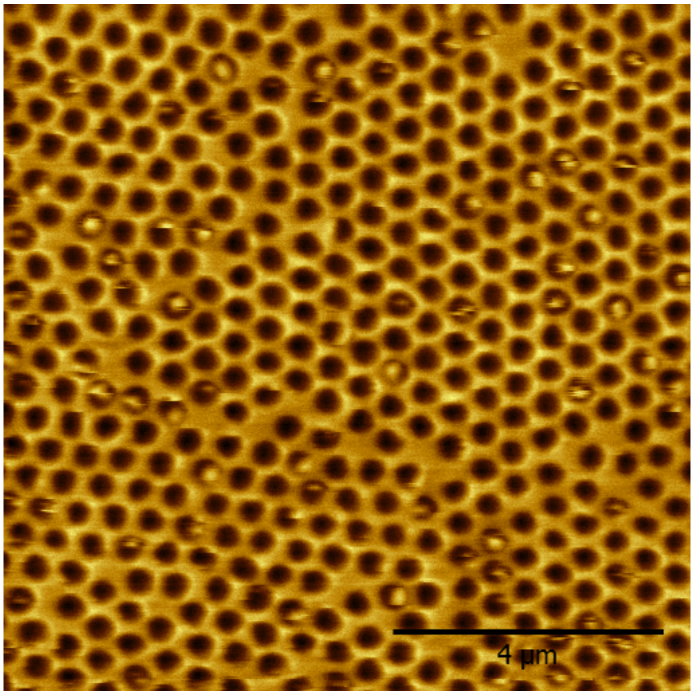

Figure S9: MFM over a larger scanning area (10 x 10 μm^2) of the sample with isolated skyrmion from Sample 1.

large number of skyrmions. As mentioned in the main text, about 20 % of the nanomagnets exhibited single skyrmions.

As discussed in the main text, to enhance the probability of observing the skyrmionic texture as predicted by the phase diagram, we analyzed a sample with lower K_{eff}, which we will call Sample 2. This sample has an effective anisotropy constant (K_{eff}) of approximately $\mathrm{K}_{\mathrm{eff}} \approx 0.01$ MJm^{-3}. To verify the occurrence of skyrmionic states in Sample 2, we imaged it using Magnetic Force Microscopy (MFM). The magnetic texture of sample 2 is shown in Figure S10a, while the topographic image is presented in Figure S10b.

Our investigations revealed various magnetic structures on the nanospheres, particularly skyrmions and skyrmioniums, highlighted by blue arrows in Figure S10a. Furthermore, we identified two skyrmion textures, marked by red arrows in Figure S10c. The corresponding topography images are presented in Figures S10b and S10d. These findings support our simulations, which show that these topological skyrmionic textures correspond to the lowest energy configurations within this diagram range, illustrated in Figure 2h. Moreover, we did not observe any nanocaps exhibiting radial or monodomain configurations, suggesting that the magnetic parameters of this sample are significantly distant from any boundaries in the phase diagram.

The results indicate that a decrease in the K_{eff} value leads to an increase in the presence of skyrmionic states. Therefore, we can conclude that the overall behavior aligns with our expectations based on the phase diagram and the ability to adjust each energy term independently.

Figure S11 presents the MFM image of sample 2 in differents area (a, b, and c). Based on this MFM images and others more we calculated the statistics of skyrmionic states for this sample how presented in Figure S11d and main text.

Figure S12 presents scans of the MFM tip in various directions, performed to assess the potential influence of its stray field on the observed magnetic textures. Given that the MFM tip used in the measurements has a high magnetic moment, its emitted field could,

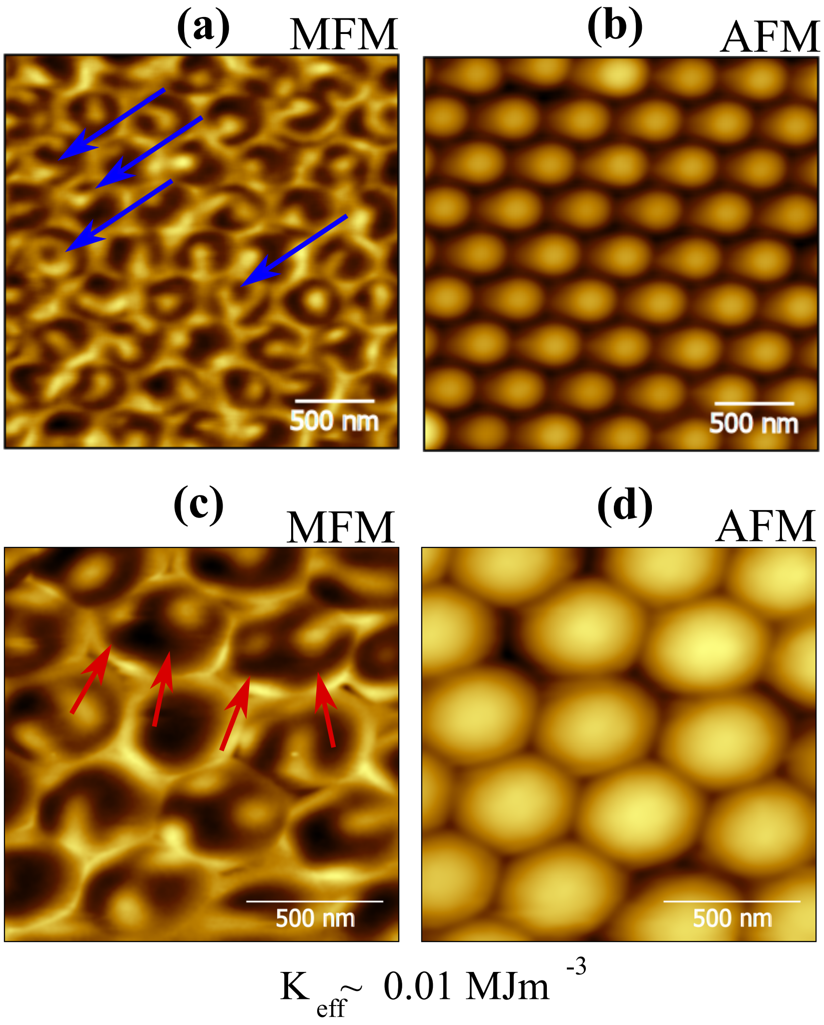

Figure S10: Magnetic (a, c) and topographic (b, d) images of the sample with $K_{\text{eff}} \approx 0.01$ MJm^{-3} on 500 nm PS. Skyrmions and skyrmionium are observed. Blue arrows indicate skyrmionium-type structures (a), and red arrows show two skyrmions on the same nanomagnet (c).

in principle, alter the magnetic configuration of the sample, potentially even inducing the formation of skyrmions. If the tip had any effect on the imaged texture, we would expect the observed structures to change when varying the scan direction. In particular, if the tip were responsible for skyrmion nucleation, skyrmions should appear randomly as the scanning orientation changes. However, we observed that when altering the scan direction, the bright spots (interpreted as skyrmions) remained stationary, and the same regions that originally exhibited skyrmions continued to do so. This consistency across different scan orientations

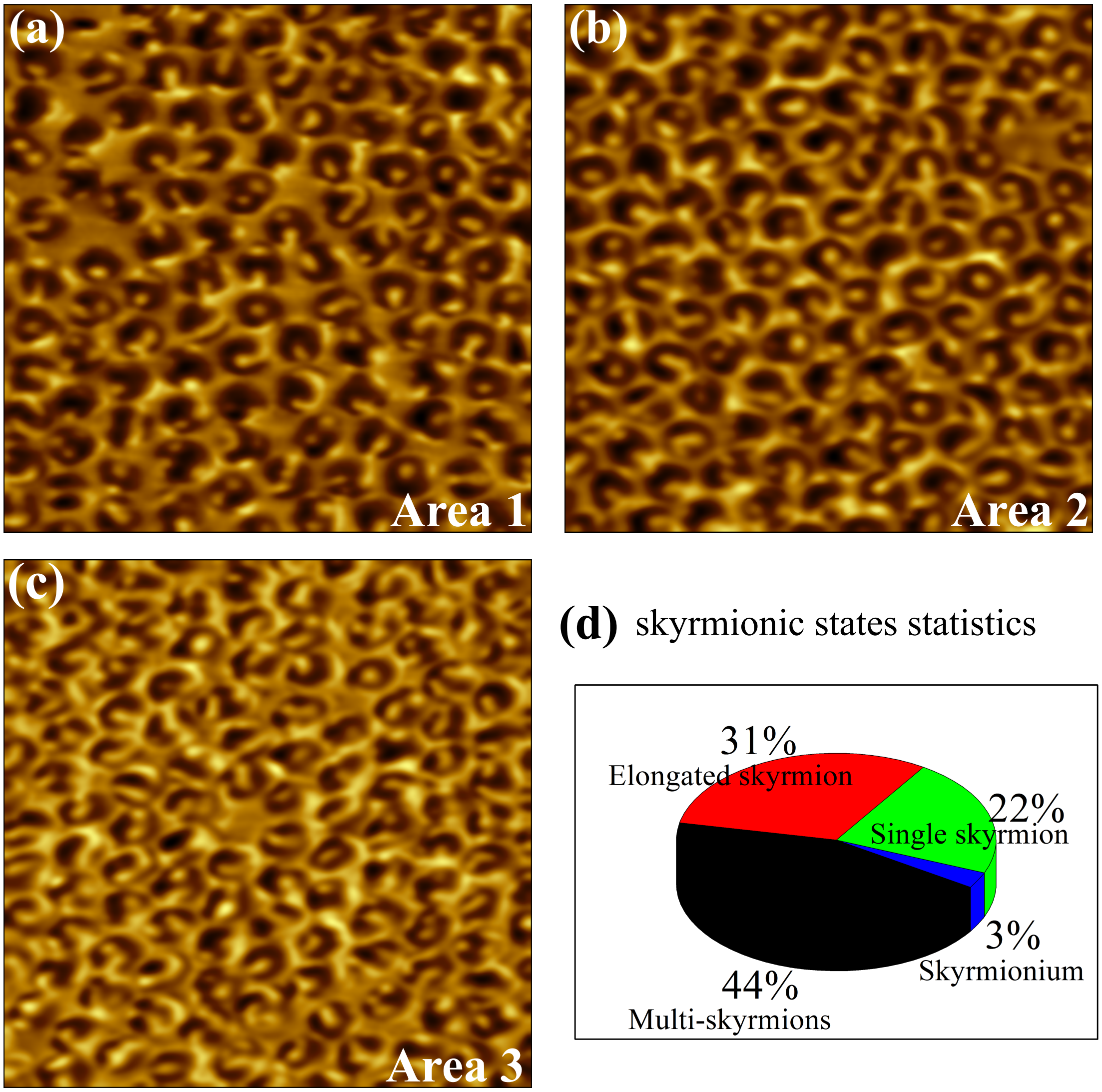

Figure S11: MFM images of sample 2 in direfferents areas to calculate the statistis of skyrmionic states presented in the main text.

indicates that the MFM tip does not influence the magnetic texture. The same conclusion holds for all observed samples, reinforcing that the skyrmionic states are intrinsic to the material and not artifacts induced by the MFM measurement.

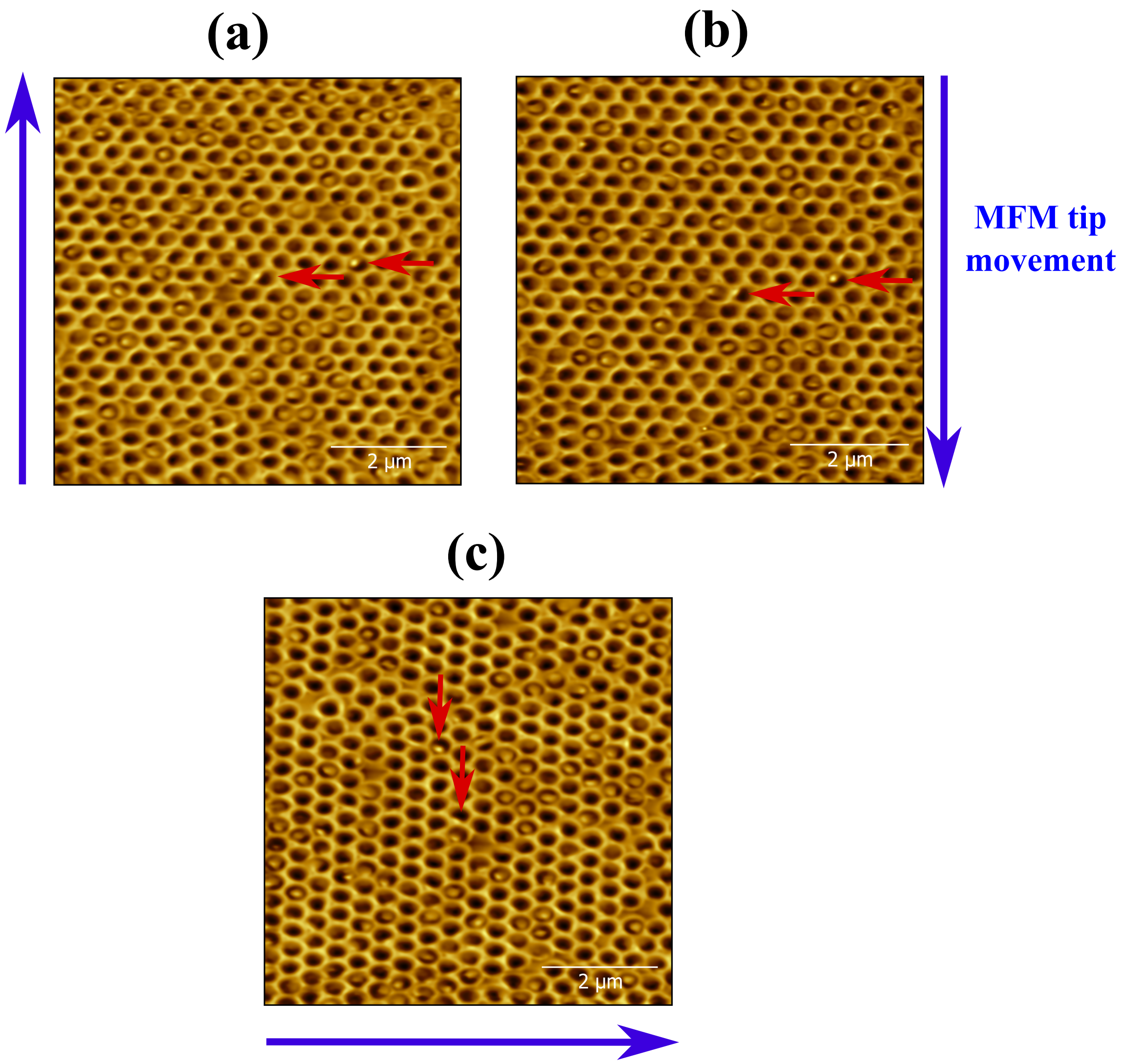

Figure S12: MFM of sample 1 with tip movement in different directions. The blue arrow indicates the scan direction.

S3.3 - Electron holography and simulated phase images

S3.3.1 - Off-axis electron holography

A Thermo Fisher Titan TEM equipped with an image-C_S corrector and an electron biprism was used at an accelerating voltage of 300kV (Ernst Ruska-Centre for Microscopy and Spectroscopy with Electrons, Forschungszentrum Jülich, Germany) to perform the off-axis electron holography shown in Figure 5. Electron holograms were acquired under field-free condi-

tions in Lorentz mode on a Gatan K2 Summit high-speed direct detection camera. A voltage of 150 V was applied to the biprism, resulting in an interference fringe spacing of ~ 2.9 nm. Stacks of 5 electron holograms (each acquired for 4s) were aligned and then averaged using the Holoview software to improve the signal to noise ratio of the reconstructed phase images. The total phase shift, $\phi(x, y)$, of the electron wave recorded perpendicular to the incident electron beam direction z has two components sensitive to (1) the electrostatic potential, ϕ_e, and; (2) the out-of-plane component of the magnetic vector potential, ϕ_m, in the specimen, as summarized by:

$$\phi(x, y) = \phi_e(x, y) + \phi_m(x, y) = C_E \int V(x, y, x) dz - \left(\frac{2\pi e}{\hbar}\right) \int A_z(x, y, x) dz \tag{S4}$$

where C_E is an interaction constant at the chosen TEM accelerating voltage, V is the electrostatic potential, e is the elementary charge of an electron and A_z is the out-of-plane magnetic vector potential. The ϕ_m is proportional to the thickness-integrated magnetic induction, B, of the sample perpendicular to the electron beam, using the relation between A and B ($B = \nabla \times A$). The ϕ_m can be isolated through acquiring two separate electron hologram sets before and after reversing the sign of A_z and ϕ_m of the sample, subtracting one ϕ from the other to eliminate the ϕ_e contribution, and dividing by two. To achieve this without applying a magnetic field, the nanocap samples were physically flipped by 180° inside the TEM using a dedicated tomographic holder.[19] The reconstructed pairs of averaged ϕ images were digitally flipped, rotated and subtracted to isolate the ϕ_m contribution, as shown in S13a-c. A circular mask is added to S13c (green circle) to isolate the magnetic contrast from artefacts introduced by incomplete ϕ unwrapping caused by large thicknesses at the nanocap edges (S13d). To create the magnetic induction maps, the ϕ_m images underwent Gaussian smoothing (S13e) and the cosine was amplified to produce magnetic phase contours, and color wheels are used to show the direction of the projected induction (S13f).

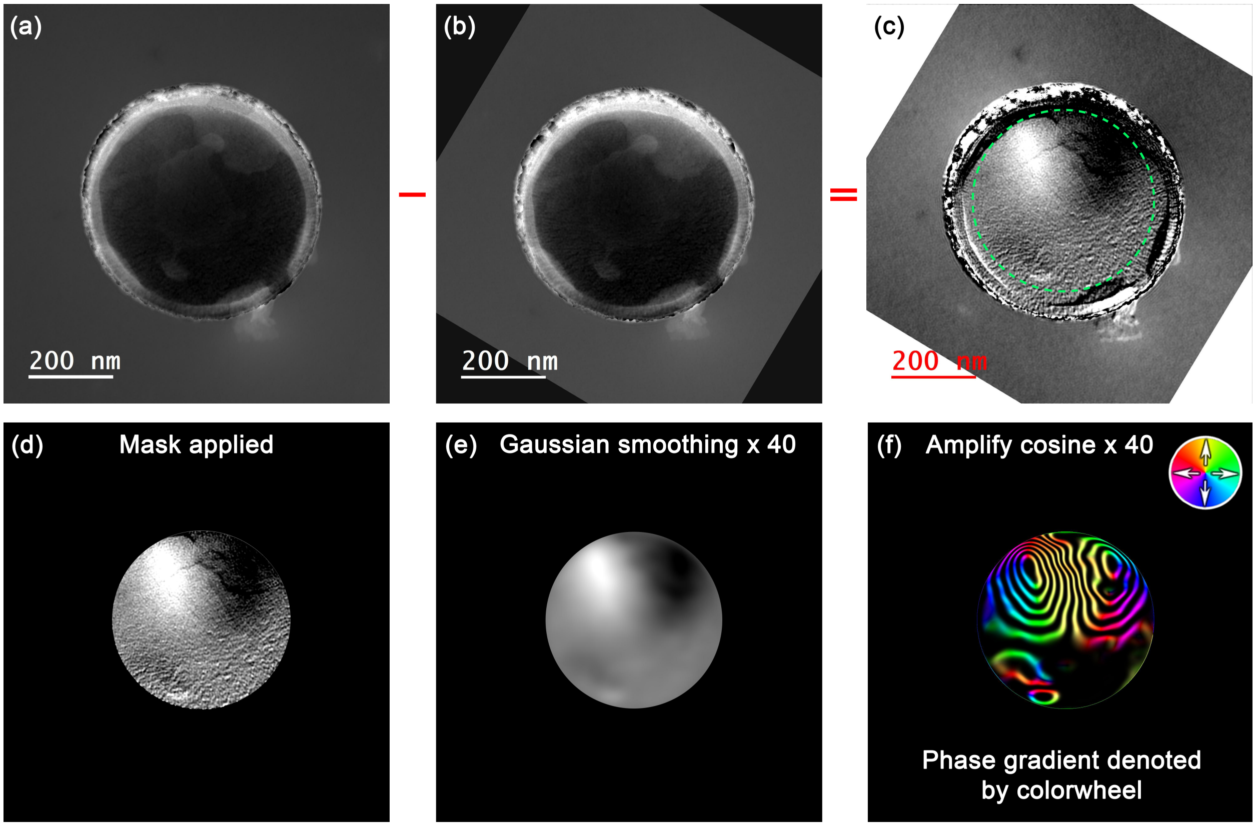

Figure S13: (a) Individual ϕ image of a nanocap from which the; (b) physically flipped by 180°, then digitally flipped and rotated ϕ image was subtracted. (c) Difference ϕ image that is divided by two to represent the ϕ_m image. (d) ϕ_m image with mask applied to remove artefacts beyond the green circle in (c). (e) Gaussian smoothing (×40) and (f) amplification of the cosine (×40) to create magnetic contours and the magnetization direction is determined by the phase gradient denoted in the colorwheel (inset).

S3.3.2 - Quantification of holography of a single skyrmion

To validate the electron holography results, a quantitative comparison with the ϕ_m expected from an idealised Néel-skyrmion is performed. The following material properties and experimental details must be known to construct the idealised skyrmion: the saturation induction of the material ($\mu_0 M_s$); the local thickness of magnetic material; the local tilt between incident electron beam and sample; and the size of the skyrmion (which changes the H contribution to the measured B). The complex nanocap morphology with both non-uniform magnetic thickness and radially varying angle of sample orientation relative to the electron beam makes direct and quantitative comparison to micromagnetic simulations challenging.

Hence, a simple model of a skyrmion in a thin film was constructed where all key parameters were matched accurately to the local environment of the skyrmion presented in Figure S10. Here, only the M_z of the skyrmion is modelled, as all Lorentz TEM methods are insensitive to the in-plane component of Néel-type textures.[20–22]

The skyrmion diameter of 195 nm was measured directly from the experimentally acquired ϕ_m image, shown again in S14a, and is defined as the distance between the minima of black contrast and maxima of white contrast, over which $M_z = 0$. Similarly, the skyrmion centre was found to be 115 nm from the centre of the nanocap (where the multilayered structure is at its thickest). The nominal structure at the nanocap centre is [Pt(1 nm)/Co(2 nm)/Pt(1 nm)]×10 and hence comprises 20 nm of magnetic Co. The thickness gradient of the nanocap was measured experimentally and a normalised thickness profile of the nanocap is shown in S14b. The black vertical line marks the location of the centre of the skyrmion, showing it is positioned over an area with a localised thickness 95% of its maximum and reduced Co thickness of 19 nm. AFM height data (S14c) was used to determine the local angle of sample orientation relative to the electron beam and was measured to be 29° at the centre of the skyrmion. M_s was measured experimentally to be 0.5 MA/m, with an associated B_s of 0.6 T. The M_z distribution of a simple skyrmion with diameter 195 nm was created in Python (S14d) using a pixel size of 0.5 nm to match the experimental data. Note that a significant background phase ramp is present in holography images of Neel skyrmions in thin films.[22] This effect is due to the uniform M_z background, however, if instead the background M is radial, this ramp should not be present due to divergent M configurations giving no contrast in Lorentz microscopy. Therefore, as the nanocaps are expected to have a radial M distribution this background ramp is not expected and is not included in the calculated phase image. Custom implementation (in Digital Micrograph) of the algorithm proposed by M. Mansuripur[23] allowed calculation of the ϕ_m from the M_z distribution (S14e) using all the above values for input tilt, B_s and thickness. Considering the radially varying parameters of the experimentally measured ϕ_m and the use of flat model of a skyrmion in

a thin film, only the centre of the simulated ϕ_m is expected to match. Line profiles were acquired from the same central area of the experimental ϕ_m and simulated ϕ_m images of the skyrmions, showing excellent quantitative correlation (S14f). A very small linear ramp (slope $\sim$ 0.001 rad/nm) was added to the calculated line profile in order to the best match to the experimental data. This means that in the real nanocap, the M must be deviating slightly from a perfectly radial contribution.

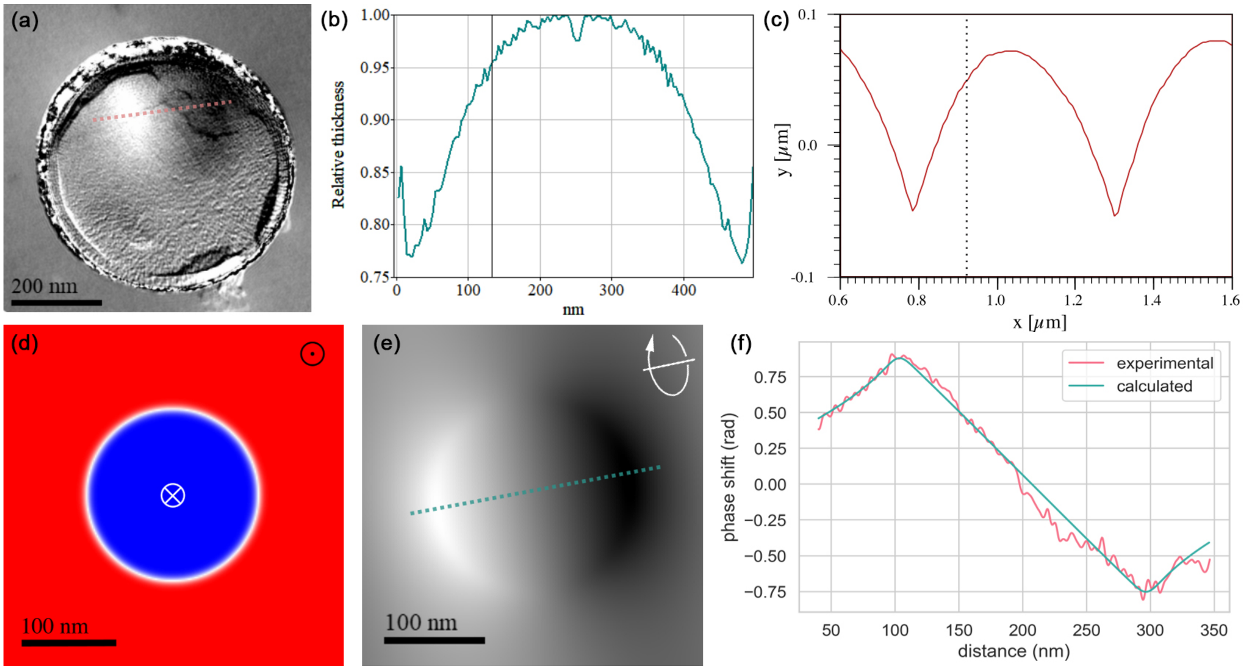

Figure S14: (a) Experimentally acquired ϕ_m image of a single skyrmion sitting towards the edge of a nanocap. (b) Normalised radial thickness variation of nanocap, where the position of skyrmion centre is marked by vertical black line. (c) AFM profile through a nanocap array, showing the position of skyrmion centre (dashed vertical line). (d) Simple model of the M_z of a skyrmion with a 195 nm diameter. (e) The calculated ϕ_m from the M_z shown in (d) with local thickness of 19 nm, 29° beam-sample angle and Ms of 0.5 MA/m. (f) Line profiles acquired through the experimental ϕ_m (dotted line in (a)) and the calculated ϕ_m images (dotted line in (e)).

S3.3.3 - Micromagnetic simulations and projected phase images.

ϕ_m images can be calculated from arbitrary magnetisation distributions, as was performed in section S3.3.2 for a simple quantitative model. For completeness, the ϕ_m images presented in S15 were also calculated from a range of magnetic textures stabilised in micromagnetic sim-

ulations of nanocaps: a single Néel-type skyrmion state, a single Bloch-type state, a double Néel-type skyrmion state, and a quadruple Néel-type skyrmion state. In these simulations, the magnetic textures primarily form in the centre of the nanocap, whilst they tend to be positioned towards the edge of the nanocap in the electron holography results. This makes direct comparison challenging as the local tilt and local thickness is inconsistent between the experiment vs micromagnetic simulation. Hence, the below images (S15) were supplied to simply show the contrast signature, and not quantitative ϕ_m values for comparison with experimental results. Furthermore, contrast in all Lorentz TEM methods is sensitive to the angle between the incident electron beam and B.[20–22] There are two key considerations regarding image contrast: (a) B which is parallel to the electron beam does not result in a ϕ_m (but contrast can be gained by introducing a tilt between the B and electron beam) and (b) the in-plane component of Néel-type walls and skyrmions do not contribute contrast with any beam-sample angle, but Bloch-type walls do contribute contrast. Since the skyrmions are located centrally within the simulated nanocaps, ϕ_m images were calculated with both 0° and 20° tilt angle of the nanocaps. The latter scenario should approximate the contrast signature if the textures are positioned at the edges of the nanocap, where there is an inherent tilt angle due to their curvature.

The Néel-type skyrmion presented in S15a is shown to be invisible at 0° tilt angle (S15b), but exhibits contrast consistent with experimental results at 20° tilt angle (S15c,d). The Bloch-type skyrmion is visible at both 0° (S15e,f) and 20° tilt angles (S15g,h), but with a slightly different signature when tilted compared to Néel-type skyrmions. Both the double (S15i) and quadruple Néel-type skyrmion states (S15m) are positioned away from the nanocap centre, and hence are visible in 0° (S15j,n) and 20° tilt angles (S15l,p) due to the nanocap curvature. Considering the contrast is due to being sensitive to the in-plane component of B provided by this curvature, the directionality of the characteristic 'black-white' lobes reflects this local tilt. This provides further evidence to confirm the skyrmionic nature of the contrast, as it is always observed in the correct orientation with respect to the local

tilt. For example, the experimental ϕ_m images of the skyrmion in S13c matches the nature of the top skyrmion in the four skyrmion state (S15n,p). In addition, there was no visible contrast at the centre of the nanocaps in all experimental holography images acquired, which suggests they are indeed Néel-type skyrmions, whereas Bloch-type skyrmions would have been easily identified, as shown in S15f.

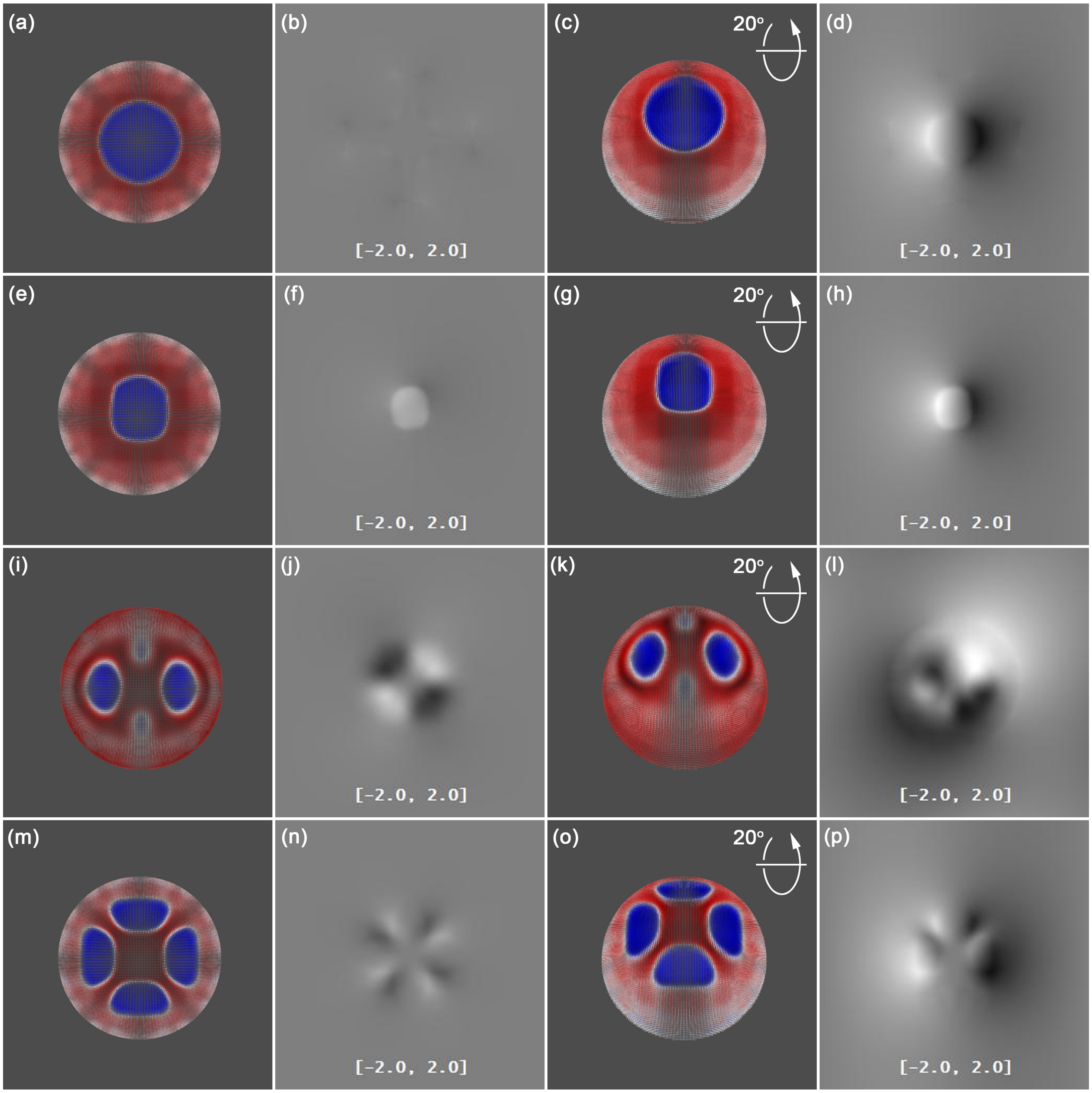

Figure S15: Comparison of micromagnetic simulations and projected ϕ_m images at 0° and 20° tilt angle. (a,c) Micromagnetic simulation; and (b,d) projected ϕ_m images of a single Néel skyrmion at (a,b) 0°; and (c,d) 20° tilt angles. (e,g) Micromagnetic simulation; and (f,h) projected ϕ_m images of single Bloch skyrmion at (e,f) 0°; and (g,h) 20° tilt angles. (i,k) Micromagnetic simulation; and (j,l) projected ϕ_m images of two Néel skyrmions at (i,j) 0°; and (k,l) 20° tilt angles. (m,o) Micromagnetic simulation; and (n,p) projected ϕ_m images of four Néel skyrmions at (m,n) 0°; and (o,p) 20° tilt angles. The simulated ϕ_m images are all on the scale of −2.0 to 2.0 radians.

References

(1) Hohenberg, P.; Kohn, W. Inhomogeneous Electron Gas. *Phys. Rev.* **1964**, *136*, B864–B871.

(2) Kohn, W.; Sham, L. J. Self-Consistent Equations Including Exchange and Correlation Effects. *Phys. Rev.* **1965**, *140*, A1133–A1138.

(3) Giannozzi, P. et al. Advanced capabilities for materials modelling with Q uantum ESPRESSO. *Journal of Physics: Condensed Matter* **2017**, *29*, 465901.

(4) Perdew, J. P.; Burke, K.; Ernzerhof, M. Generalized Gradient Approximation Made Simple. *Phys. Rev. Lett.* **1996**, *77*, 3865–3868.

(5) Kresse, G.; Joubert, D. From ultrasoft pseudopotentials to the projector augmented-wave method. *Phys. Rev. B* **1999**, *59*, 1758–1775.

(6) Liechtenstein, A. I.; Katsnelson, M. I.; Antropov, V. P.; Gubanov, V. A. Local spin density functional approach to the theory of exchange interactions in ferromagnetic metals and alloys. *Journal of Magnetism and Magnetic Materials* **1987**, *67*, 65–74.

(7) Agapito, L. A.; Ferretti, A.; Calzolari, A.; Curtarolo, S.; Buongiorno Nardelli, M. Effective and accurate representation of extended Bloch states on finite Hilbert spaces. *Phys. Rev. B* **2013**, *88*, 165127.

(8) Agapito, L. A.; Curtarolo, S.; Buongiorno Nardelli, M. Reformulation of DFT + U as a Pseudohybrid Hubbard Density Functional for Accelerated Materials Discovery. *Phys. Rev. X* **2015**, *5*, 011006.

(9) Agapito, L. A.; Fornari, M.; Ceresoli, D.; Ferretti, A.; Curtarolo, S.; Buongiorno Nardelli, M. Accurate tight-binding Hamiltonians for two-dimensional and layered materials. *Phys. Rev. B* **2016**, *93*, 125137.

(10) Buongiorno Nardelli, M.; Cerasoli, F. T.; Costa, M.; Curtarolo, S.; Gennaro, R. D.; Fornari, M.; Liyanage, L.; Supka, A. R.; Wang, H. PAOFLOW: A utility to construct and operate on ab initio Hamiltonians from the projections of electronic wavefunctions on atomic orbital bases, including characterization of topological materials. *Computational Materials Science* **2018**, *143*, 462 – 472.

(11) Cerasoli, F. T.; Supka, A. R.; Jayaraj, A.; Costa, M.; Siloi, I.; Sławińska, J.; Curtarolo, S.; Fornari, M.; Ceresoli, D.; Buongiorno Nardelli, M. Advanced modeling of materials with PAOFLOW 2.0: New features and software design. *Computational Materials Science* **2021**, *200*, 110828.

(12) Costa, M.; Costa, A. T.; Freitas, W. A.; Schmidt, T. M.; Buongiorno Nardelli, M.; Fazzio, A. Controlling Topological States in Topological/Normal Insulator Heterostructures. *ACS Omega* **2018**, *3*, 15900–15906.

(13) Costa, M.; Peres, N. M. R.; Fernández-Rossier, J.; Costa, A. T. Nonreciprocal magnons in a two-dimensional crystal with out-of-plane magnetization. *Phys. Rev. B* **2020**, *102*, 014450.

(14) Vansteenkiste, A. The design and verification of `MuMax3`. *AIP Adv.* **2014**, *4*, 107133.

(15) Soares, M. M.; de Biasi, E.; Coelho, L. N.; dos Santos, M. C.; de Menezes, F. S.; Knobel, M.; Sampaio, L. C.; ; Garcia, F. Magnetic vortices in tridimensional nanomagnetic caps observed using transmission electron microscopy and magnetic force microscopy. *Phys. Rev. B* **2005**, *77*, 224405.

(16) Jalil, W.; Dugato, D.; Almeida, T.; Cooper, D.; Garcia, F. Self-supported `3D` vortex texture in curved magnets. *Journal of Physics D - Applied Physics* **2023**, *56*, 385001.

(17) Brandão, J.; Dugato, D. A.; Seeger, R. L.; Denardin, J. C.; Mori, T. J. A.; Cezar, J. C. Observation of magnetic skyrmions in unpatterned symmetric multilayers at room temperature and zero magnetic field. *Sci. Rep.* **2019**, *9*, 4144.

(18) Dugato, D. A.; Brandão, J.; Béron, F.; da Silva, R. B.; Flewett, S.; Shapiro, D. A.; Cezar, J. C.; Dorneles, L. S.; Mori, T. J. Proximity induced moment at Pt/Co interfaces and isolated skyrmion bubble stabilization at zero magnetic field. *J. Mag. Mag. Mat.* **2022**, *566*, 170305.

(19) Diehle, P.; Kovács, A.; Duden, T.; Speen, R.; Žagar Soderžnik, K.; Dunin-Borkowski, R. A cartridge-based turning specimen holder with wireless tilt angle measurement for magnetic induction mapping in the transmission electron microscope. *Ultramicroscopy* **2021**, *220*, 113098.

(20) Fallon, K.; McVitie, S.; Legrand, W.; Ajejas, F.; Maccariello, D.; Collin, S.; Cros, V.; Reyren, N. Quantitative imaging of hybrid chiral spin textures in magnetic multilayer systems by Lorentz microscopy. *Physical Review B* **2019**, *100*, 214431.

(21) McVitie, S.; Hughes, S.; Fallon, K.; McFadzean, S.; McGrouther, D.; Krajnak, M.; Legrand, W.; Maccariello, D.; Collin, S.; Garcia, K.; Reyren, N.; Cros, V.; Fert, A.; Zeissler, K.; Marrows, C. H. A transmission electron microscope study of Néel skyrmion magnetic textures in multilayer thin film systems with large interfacial chiral interaction. *Scientific Reports* **2018**, *8*, 5703.

(22) Denneulin, T.; Caron, J.; Hoffmann, M. J.; Lin, M.; Tan, H.; Kovács, A.; Blügel, S.; Dunin-Borkowski, R. E. Off-axis electron holography of Néel-type skyrmions in multilayers of heavy metals and ferromagnets. *Ultramicroscopy* **2021**, *220*, 113155.

(23) Mansuripur, M. Computation of electron diffraction patterns in Lorentz electron microscopy of thin magnetic films. *Journal of Applied Physics* **1991**, *69*, 2455.
